# Artificial Intelligence‐Based Body Composition Analysis Reveals Sex‐Specific Prognostic Markers and Their Clinical Value in Gastric Cancer: A Multicenter Study

**DOI:** 10.1002/advs.75859

**Published:** 2026-06-01

**Authors:** Tianxiang Li, Tingting Liu, Qian Yang, Zhiqing Zhang, Xiaofang Guo, Xiang Wang, Yihan Zhang, Limin Ge, Yu Su, Wenjun Wu, Weihua Guo, Zheng Wang, Lian Yang, Yanfeng Niu

**Affiliations:** ^1^ Department of Radiology Union Hospital Tongji Medical College Huazhong University of Science and Technology Wuhan China; ^2^ Hubei Provincial Clinical Research Center For Precision Radiology & Interventional Medicine Wuhan China; ^3^ Hubei Key Laboratory of Molecular Imaging Wuhan China; ^4^ Department of Radiology Hubei Cancer Hospital Tongji Medical College Huazhong University of Science and Technology Wuhan China; ^5^ Department of Thoracic Surgery The First Affiliated Hospital of Wenzhou Medical University Wenzhou Zhejiang China; ^6^ Department of Clinical Laboratory Union Hospital Tongji Medical College Huazhong University of Science and Technology Wuhan China; ^7^ Department of Gastrointestinal Surgery Union Hospital Tongji Medical College Huazhong University of Science and Technology Wuhan China; ^8^ Hubei Provincial Engineering Research Center of Clinical Laboratory and Active Health Smart Equipment Wuhan China; ^9^ Hubei Key Laboratory of Regenerative Medicine and Multi‐disciplinary Translational Research Wuhan China; ^10^ Key Laboratory of Biological Targeted Therapy Huazhong University of Science and Technology Ministry of Education Wuhan Hubei China; ^11^ Clinical Research Center of Minimally Invasive Surgery Union Hospital Tongji Medical College Huazhong University of Science and Technology Wuhan China; ^12^ Research Center for Tissue Engineering and Regenerative Medicine Union Hospital Tongji Medical College Huazhong University of Science and Technology Wuhan China

**Keywords:** Artificial intelligence, Deep learning, Gastric Cancer

## Abstract

**Background and aims**: Conventional body composition assessment fails to capture its multidimensional complexity in gastric cancer (GC). This study aimed to systematically evaluate multidimensional body composition and its clinical relevance in GC.

**Methods**: 1196 GC patients and 983 healthy controls were retrospectively enrolled. Body composition was segmented using nnU‐Net. Propensity score matching was used to compare body composition differences between patients and healthy controls. Prognostic value was assessed across treatment cohorts. TCGA pathological and transcriptomic data were integrated for exploratory analyses. Sex‐specific prognostic models were developed and externally validated.

**Results**: AI‐drived body compositions parameters showed sex‐specific associations with survival. Higher muscle/fat area/index and lower fat density correlated with longer survival in surgical patients (L1 muscle area: HR = 0.438, ad *p* = 0.001). In ICI‐treated females, higher subcutaneous fat area (SFA) improved survival (L2SFA: HR = 0.972, ad *p* = 0.045). Potential associations were observed between body composition and tumor microenvironmental features, including stromal composition, EMT‐related pathways, and immune infiltration. Sex‐specific prognostic models achieved C‐indices of 0.723 (males) and 0.705 (females) in test cohorts, outperforming conventional predictors.

**Conclusion**: Multidimensional body composition is associated with prognosis in GC and may serve as a complementary biomarker for risk stratification.

## Graphical abstract

This study applied AI to quantify multidimensional body composition from CT images in gastric cancer and healthy controls. Distinct sex‐specific patterns and disease‐related alterations were identified and were associated with survival. Higher muscle and fat measures were linked to improved outcomes. Integrating these features with clinical variables improved prognostic models and supports body composition as a biomarker.

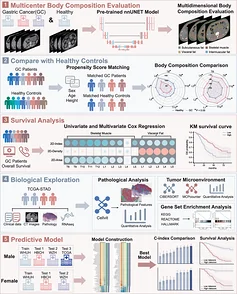

## 1 Introduction

Gastric cancer (GC) remains a major global health burden, with high incidence and mortality rates, particularly in East Asia [1]. Despite significant advances in surgery, chemotherapy, targeted therapy, and immunotherapy, the overall survival (OS) rate for advanced GC patients remains unsatisfactory, with considerable interindividual prognostic heterogeneity [2, 3]. Current prognostic assessment relies largely on clinicopathological factors such as TNM stage, histological type, and tumor marker levels, while these parameters incompletely capture interindividual variability and offer limited predictive power for treatment response and long‐term outcomes [2, 4]. Consequently, identifying novel prognostic biomarkers that reflect patients’ comprehensive physiological status and are independent of conventional factors has become a key focus in precision oncology for GC. Malnutrition and metabolic dysregulation are common in GC patients and are increasingly recognized as key determinants of disease progression and therapeutic response [5, 6]. As a core measure of muscle, fat, and other tissue distribution and quality, body composition not only influences patients’ performance status and treatment tolerance but may also participate in shaping the tumor microenvironment through the modulation of inflammatory, immune, and endocrine pathways [7, 8, 9].

Previous studies majorly relied on body mass index (BMI) or single‐slice skeletal muscle area for assessing body composition in cancer patients [10, 11]. However, these metrics frequently overlook the spatial heterogeneity and three‐dimensional volumetric characteristics of body composition, failing to comprehensively reflect complex metabolic status. Recent advances in artificial intelligence have enabled automated, high‐throughput quantification of body composition from computed tomography (CT) images, providing a more comprehensive and objective assessment. Using AI‐assisted body composition quantification, multiple studies have demonstrated that sarcopenia is closely associated with poor prognosis in various solid tumors [12, 13]. In GC, some studies suggest that low skeletal muscle mass and low visceral fat predicts worse survival outcomes [14, 15]. While prior studies have provided important insights into the prognostic relevance of body composition in cancer, most have focused on single‐level measurements (predominantly L3) or single‐center cohorts, with limited integration of multidimensional features and external validation. In addition, comparisons with healthy populations remain relatively underexplored. These gaps highlight the need for more comprehensive and systematically validated approaches. More importantly, the underlying biological mechanisms connecting body composition alterations to prognosis in GC patients, particularly their intrinsic relationship with the tumor microenvironment, have yet to be elucidated.

Given this, this study established a health screening cohort and a multicenter GC cohort to systematically investigate the differences in body composition between gastric cancer patients and healthy individuals, as well as their clinical implications. By integrating automated CT analysis with clinical outcome data, we first evaluated the prognostic implications of multidimensional body composition. Furthermore, by incorporating histopathological and transcriptomic data, we explored its potential associations with tumor microenvironmental features. Furthermore, we constructed and externally validated sex‐specific prognostic prediction models that incorporate multidimensional body composition and clinical variables, thereby offering a practical tool for individualized risk stratification. This work not only provides robust imaging biomarkers for prognosis but also lays a foundation for exploring potential therapeutic strategies targeting body composition in GC management.

## 2 Methods

### 2.1 Patients

**FIGURE 1:**
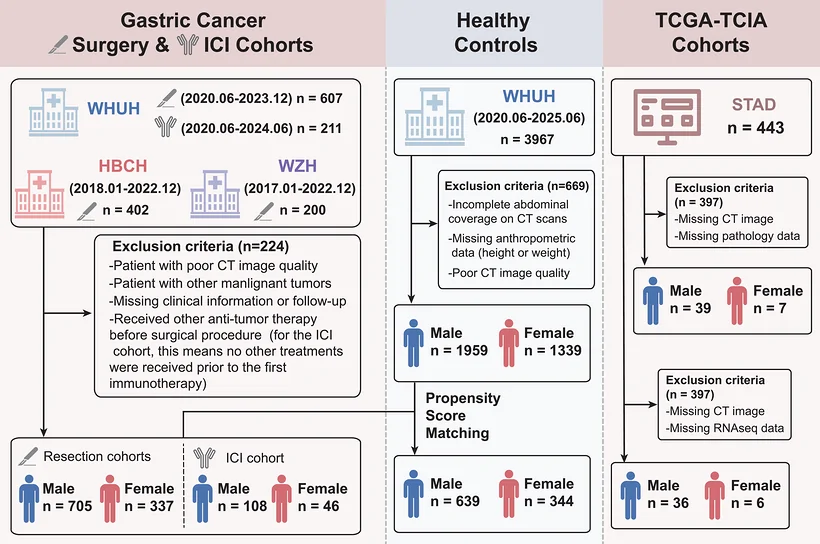
Flowchart of patient inclusion and exclusion.

This study retrospectively analyzed four GC cohorts from three hospitals across China, including patients receiving surgical resection or ICI treatments, and one health control cohort from Union Hospital, Tongji Medical College, Huazhong University of Science and Technology (WHUH). Inclusion criteria for the GC patients were as follows: (1) absence of other primary malignant tumors; (2) histologically confirmed diagnosis of GC; (3) availability of abdominal CT images obtained within 30 days prior to the initiation of first‐line treatment (either surgical resection or immunotherapy); (4) availability of complete clinicopathological and follow‐up data. Exclusion criteria were: (1) incomplete clinical or follow‐up data; (2) poor CT image quality or unsuitability for assessment; (3) receipt of any anti‐tumor treatments prior to surgery (for the ICI cohort, this means no other treatments were received prior to the first immunotherapy). For the healthy control cohort, all participants were individuals undergoing routine health examinations at the physical examination center, and abdominal CT scans were performed as part of standard screening rather than for any specific clinical indication or disease‐related symptoms. Inclusion criteria were: (1) availability of abdominal CT imaging; (2) no known respiratory, cardiovascular, endocrine, neurological, urinary, or immune system diseases; (3) no history of malignancy or current anti‐cancer treatment; (4) no significant space‐occupying or active lesions detected on imaging or routine physical examination. Exclusion criteria were: (1) poor CT image quality, with severe respiratory artifacts that obscured the boundary between muscle and adipose tissue; (2) missing height and weight data. In total, 1196 GC patients and 983 healthy controls were included in the final analysis. Patients from each participating center were consecutively enrolled during predefined study periods, and detailed recruitment timelines are provided in Figure 1. This study was conducted in accordance with the principles of the Declaration of Helsinki, and its reporting followed the Transparent Reporting of a multivariable prediction model for Individual Prognosis or Diagnosis (TRIPOD) guidelines [16]. This study was approved by the Ethics Committees of WHUH, Hubei Cancer Hospital (HBCH), and the First Affiliated Hospital of Wenzhou Medical University (WZH). All patient information was anonymized, and written informed consent was waived due to the retrospective nature of the study. Overall survival (OS) was used as the primary endpoint and defined as the time from initiation of first‐line therapy to death from any cause. Follow‐up data were primarily obtained from routine clinical electronic records, with additional telephone follow‐up conducted when necessary to ensure completeness of outcome information. Patients were routinely followed every 6 months during the first 2 years after treatment and annually thereafter. The median follow‐up duration for GC patients in this study was 34 months.

### 2.2 Automated Measurement of Multidimensional Body Composition With Pre‐Trained Deep Learning Model

We developed an automated framework for multidimensional body composition analysis, extracting 16 two‐dimensional (2D) and three‐dimensional (3D) parameters from routine CT images. A pre‐trained nnU‐Net model was employed to segment thoracic and lumbar vertebrae, identify all visible vertebral bodies, and extract their 3D coordinates (X, Y, Z) [17, 18]. In parallel, subcutaneous fat, skeletal muscle, intermuscular fat, and visceral fat were precisely segmented. Based on the Z‐axis range from the superior border of L1 to the inferior border of L5, segmented muscle and fat tissues were cropped to calculate volumetric measurements for each component. Single‐slice segmentation at the mid‐vertebral level was subsequently used to derive cross‐sectional areas (2D‐Area), which were normalized by height to obtain body composition indices (2D‐Index). Tissue densities were calculated by overlaying segmentation masks onto the original CT images (2D‐Density). In total, 16 distinct body composition features were quantified. Detailed calculation formulas and the measurement workflow are provided in Table S1. Considering that regions above T7 are susceptible to variations in patient arm positioning and scan coverage (Figure S1), body composition analysis in this study was restricted to vertebral levels T8 and below. All segmentation inference and script execution were performed on a Linux machine (Ubuntu 22.04) equipped with an NVIDIA 4090 GPU.

To assess the reliability of this body composition segmentation algorithm, 50 patients were randomly selected for validation. An abdominal radiologist with 5 years of experience performed manual measurements of subcutaneous fat area (SFA), visceral fat area (VFA), skeletal muscle area (SMA), and intermuscular fat area (IMFA) at the L3 level. Results showed high consistency between AI‐based automated measurements and manual annotations (Intraclass correlation coefficient [ICC] > 0.89) (Figure S2). Furthermore, the distributions of subcutaneous fat volume (SFV), visceral fat volume (VFV), skeletal muscle volume (SMV), and intermuscular fat volume (IMFV) were similar across the four GC cohorts (Figure S3).

### 2.3 Analysis of Pathological Data

Patients with both CT images and whole‐slide images (WSI) of hematoxylin and eosin (H&E)‐stained pathological sections were selected from the TCGA cohort. Multidimensional body composition was extracted from CT images using the aforementioned method. Tumor regions on WSIs were manually annotated and segmented using QuPath software (v0.6.0). A pretrained CellViT model based on the PanNuke dataset was employed for automated nucleus recognition and classification into the following categories: Neoplastic cell, Inflammatory cell, Dead cell, Epithelial cell, and Connective cell [19]. To quantitatively evaluate the spatial distribution characteristics of immune cells within the tumor regions, a custom Groovy script was developed on the QuPath platform to automatically extract multiple spatial pathological parameters. Specifically, the script first calculates the actual physical dimensions of the tumor area based on pixel calibration information, then efficiently computes spatial relationships between cells using a gridded spatial indexing algorithm, and finally derives a set of quantitative metrics including cell density, spatial distance, tumor‐immune colocalization, and spatial aggregation. Detailed definitions and calculation methods for these metrics are provided in Table S2. For body composition parameters that remained significant in the multivariate Cox regression analysis, their correlations with pathological parameters were analyzed using age‐adjusted correlation analysis. Patients were stratified into high and low groups based on the median value of each significant body composition parameter, and subsequent analyses compared pathological parameters between these two groups.

### 2.4 Analysis of Transcriptomic Data

Patients with both CT images and RNA‐seq data were selected from the TCGA cohort. Body composition information was obtained using the same methodology described previously. The CIBERSORT algorithm and MCP‐counter algorithm were employed to estimate the relative abundance of tumor‐infiltrating immune cell populations and cytotoxicity scores, respectively, in order to explore potential associations between multidimensional body composition and intratumoral immune infiltration [20, 21]. Additionally, age‐adjusted differential gene expression analysis was performed using raw count data and the DESeq2 package, followed by Gene Set Enrichment Analysis (GSEA) based on the Kyoto Encyclopedia of Genes and Genomes (KEGG), Reactome, and Hallmark gene sets to identify pathways potentially associated with different body composition profiles.

### 2.5 Model Construction

Based on clinical indicators and body composition, this study constructed prognostic prediction models separately for males and females. Model construction was primarily based on the surgical cohorts, given its relatively large sample size, which is sufficient to support machine learning model training, as well as the availability of multiple independent external validation cohorts. Using WHUH as the training set, HBCH and WZH as test sets, and additionally incorporating TCGA as a test set for the male cohort (n = 39). The variables included in model construction comprised key clinical factors with multidimensional body composition features derived from CT images. As height information was unavailable in the TCGA dataset, BMI and body composition indices could not be calculated; therefore, only area‐ and density‐based 2D features were incorporated for model construction to ensure consistency across cohorts. A detailed list of included features was provided in Table S3. Missing data were handled using multiple imputation via the mice package in R. Model development and hyperparameter optimization were strictly confined to the training set (WHUH) using a Leave‐One‐Out Cross‐Validation (LOOCV) framework. A total of 117 prediction model variants were constructed by combining multiple algorithms, including random survival forest (RSF), elastic net (Enet), Lasso, Ridge, stepwise Cox regression (StepCox), CoxBoost, partial least squares regression for Cox (plsRcox), supervised principal components (SuperPC), generalized boosted regression modeling (GBM), and survival support vector machine (survival‐SVM). The external cohorts (HBCH, WZH, and TCGA) were not involved in any stage of model training or hyperparameter tuning. Instead, these cohorts were used solely for independent validation. For each model, the Harrell's concordance index (C‐index) was calculated across all external validation datasets, and the model with the highest average C‐index was selected as the main model. When multiple models demonstrated comparable discriminative performance, preference was given to those exhibiting more stable performance across individual validation cohorts (particularly with less performance degradation in the worst‐performing cohort), as well as to models with simpler and more interpretable structures.

### 2.6 Statistical Analysis

Statistical analyses and data visualization were performed using R (v4.5.0), Python (v3.12), and Slicer (v5.8.1). Continuous variables are presented as mean ± standard deviation (SD) or median (Interquartile Range, IQR), while categorical variables are reported as frequency (percentage). Intraclass correlation coefficient (ICC) and Bland‐Altman plots were used to assess agreement between manual and AI‐based body composition measurements. Propensity score matching (PSM) was performed using 1:1 nearest neighbor matching with a caliper width of 0.2 to balance baseline characteristics between GC patients and healthy controls. Differences in continuous variables were analyzed using the Wilcoxon rank‐sum test or Student's t‐test, while categorical variables were compared using the chi‐square test or Fisher's exact test. Partial Spearman correlation analysis controlling for age was used to assess associations between body composition parameters with pathological parameters. Kaplan‐Meier survival curves and the log‐rank test were used to assess survival differences between groups. For continuous variables, the optimal cutoff value was determined using the surv_cutpoint function in the R package survminer. Proportional hazards assumptions were tested to ensure validity for Cox regression analysis. Cox proportional hazards regression was used to evaluate prognostic significance, and proportional hazards assumptions were verified prior to model fitting. To address the issue of multiple comparisons arising from the evaluation of numerous body composition parameters, *p* values were adjusted using Benjamini‐Hochberg method (ad *p* value). Separate multivariable Cox models were constructed for each parameter, with each model including a single body composition variable along with predefined clinical factors, to assess its independent prognostic value while minimizing multicollinearity. Hazard ratios (HRs) are reported with corresponding 95% confidence intervals. All statistical tests were two‐sided, and *p* values < 0.05 were considered statistically significant.

## 3 Results

### 3.1 Baseline Characteristics

A total of 1196 GC patients from four cohorts were included in this study, comprising 1042 patients in the surgical cohort and 154 patients in the ICI cohort (Table 1). The overall patient population consisted of 813 males (68.0%) and 383 females (32.0%). Tumor stage distribution differed significantly between treatment groups: the surgical cohort predominantly composed of patients with stage I‐III disease (96.0%), whereas the ICI cohort was largely consisted of patients with stage IV disease (89.6%). Additionally, a control cohort of 3298 healthy individuals was included (Table S4), consisting of 1959 males (59.4%) and 1339 females (40.6%). To minimize confounding, PSM was performed separately for males and females based on age and height. This process ultimately matched 639 male and 344 female pairs of healthy controls and gastric cancer patients. The baseline characteristics of all cohorts after matching are detailed in Tables S5 and S6.

**TABLE 1:** Baseline information for GC patients.

**Characteristics**	**WHUH**	**HBCH**	**WZH**	**iWHUH**
N	466	388	188	154
Age, median (IQR)	60 (54, 67)	60 (53, 67)	66 (57, 74)	62 (55, 69)
Sex, n (%)				
Male	299 (64.2%)	270 (69.6%)	136 (72.3%)	108 (70.1%)
Female	167 (35.8%)	118 (30.4%)	52 (27.7%)	46 (29.9%)
Height, median (IQR)	1.67 (1.6, 1.7)	1.67 (1.6, 1.7)	1.65 (1.6, 1.7)	1.67 (1.62, 1.7)
Weight, median (IQR)	61 (55, 70)	61.5 (55, 69)	60 (52, 68.5)	60 (53.58, 66)
BMI, median (IQR)	22.41 (20.31, 24.8)	22.10 (20.70, 24.50)	21.99 (20.07, 23.69)	21.505 (19.74, 23.41)
Grade, n (%)				
Poorly differentiated	291 (62.4%)	310 (79.9%)	134 (71.3%)	98 (63.6%)
Moderately differentiated	22 (4.7%)	14 (3.6%)	11 (5.9%)	0 (0%)
Well differentiated	122 (26.2%)	64 (16.5%)	35 (18.6%)	1 (0.6%)
Undifferentiated	24 (5.2%)	0 (0%)	0 (0%)	1 (0.6%)
Missing data	7 (1.5%)	0 (0%)	8 (4.2%)	54 (35.2%)
Stage, n (%)				
I	172 (36.9%)	76 (19.6%)	57 (30.3%)	0 (0%)
II	114 (24.5%)	105 (27.1%)	36 (19.1%)	0 (0%)
III	165 (35.4%)	188 (48.5%)	87 (46.3%)	3 (1.9%)
IV	2 (0.4%)	19 (4.9%)	8 (4.3%)	138 (89.6%)
Missing data	13 (2.8%)	0 (0%)	0 (0%)	13 (8.4%)
CEA, median (IQR)	2 (1.4, 3.3)	2.05 (1.06, 3.19)	2.58 (1.47, 4.49)	3.55 (1.72, 20.17)
CA19‐9, median (IQR)	6.35 (3, 12.45)	8.87 (5.25, 17.84)	11.28 (5.785, 19.85)	16.93 (5.1, 268.8)
Her2, n (%)				
0	331 (71.0%)	272 (70.1%)	146 (77.7%)	71 (46.1%)
1	17 (3.7%)	28 (7.2%)	14 (7.4%)	17 (11.0%)
Missing data/Uncertain	118 (25.3%)	88 (22.7%)	28 (14.9%)	66 (42.9%)
PDL1, n (%)				
CPS ≥ 10	∖	∖	∖	22 (14.3%)
CPS: 1–10	∖	∖	∖	40 (25.9%)
CPS < 1	∖	∖	∖	22 (14.3%)
Missing data	∖	∖	∖	70 (45.5%)

*Note*. WHUH: Wuhan Union Hospital, HBCH: Hubei Cancer Hospital, WZH: the First Affiliated Hospital of Wenzhou Medical University, iWHUH: immunotherapy cohort of WHUH, BMI: Body mass index, CEA: Carcinoembryonic Antigen, CA199: Carbohydrate antigen199, CPS: Combined Positive Score.

### 3.2 Distribution of Body Composition in GC Patients

**FIGURE 2:**
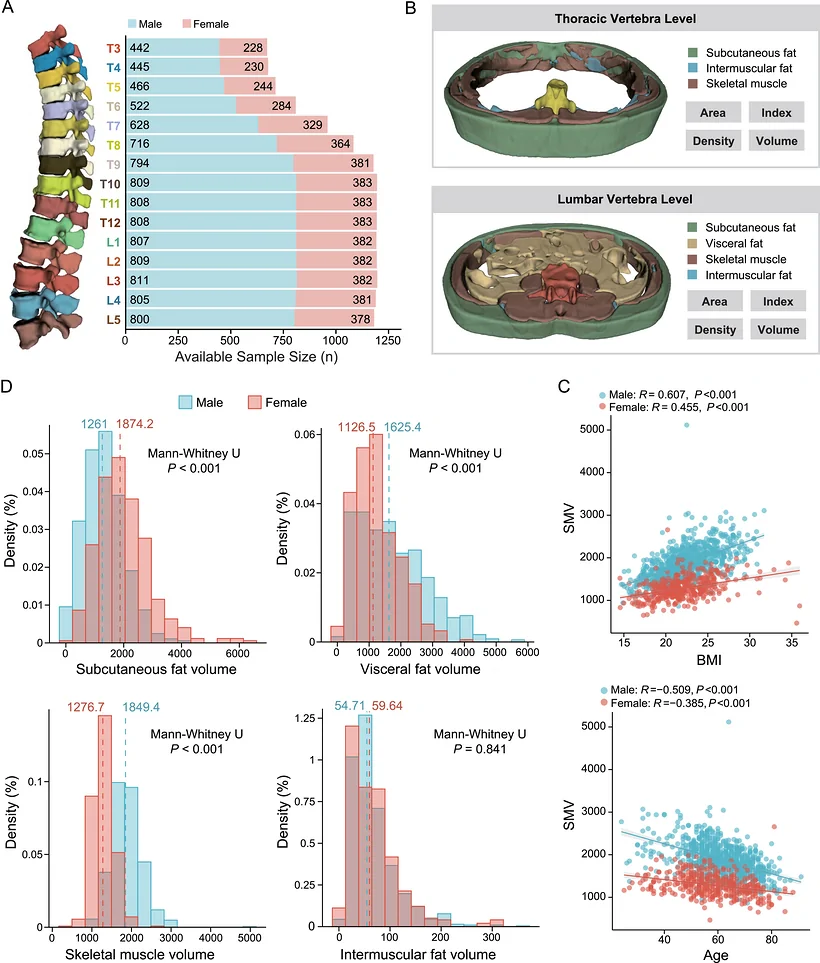
Distribution of sample size and body composition parameters in GC patients. (A) Available sample size (n) of patients at each vertebral level for male and female cohorts. (B) Schematic representation of body composition analyzed at the thoracic and lumbar vertebra level. (C) Comparison of subcutaneous fat volume (SFV), skeletal muscle volume (SMV), visceral fat volume (VFV) and intermuscular fat volume (IMFV) between males and females. (D) Analysis of the correlation of SMV with age and BMI.

Due to variations in abdominal CT scan coverage among patients, we first analyzed the sample size available at each vertebral level among 1196 GC patients (Figure 2A). The study focused on four body composition components: subcutaneous fat, intermuscular fat, skeletal muscle, and visceral fat. The 2D parameters included the cross‐sectional area, density, and height‐normalized indices at specific vertebral levels, while the 3D parameters comprised the volumetric measurements of each component within the L1‐L5 vertebral range (Figure 2B, Table S1). Analysis of the 3D volumetric data revealed that male patients had lower volumes of subcutaneous fat and intermuscular fat, but higher volumes of skeletal muscle and visceral fat compared with female patients (Figure 2C). Further analysis demonstrated that SFV, SMV, IMFV, and VFV were all significantly positively correlated with BMI in both males and females (Figure 2D, Figure S4A). Additionally, in male patients, SFV and SMV showed significant negative correlations with age, whereas IMFV exhibited a significant positive correlation with age. Similar trends were observed in female patients (Figure 2D, Figure S4B). We also analyzed correlations among multidimensional body composition parameters. At the same vertebral level, components of the same type, such as SMA, SMD, and SMI, exhibit a certain degree of correlation, and similar among various fat parameters (Figures S5 and S6). Furthermore, for each specific parameter type, exceptionally strong correlations were found across different vertebral levels (Figures S7 and S8).

### 3.3 GC Patients Exhibit Sex‐Specific Body Composition Alterations Compared to Healthy Controls

**FIGURE 3:**
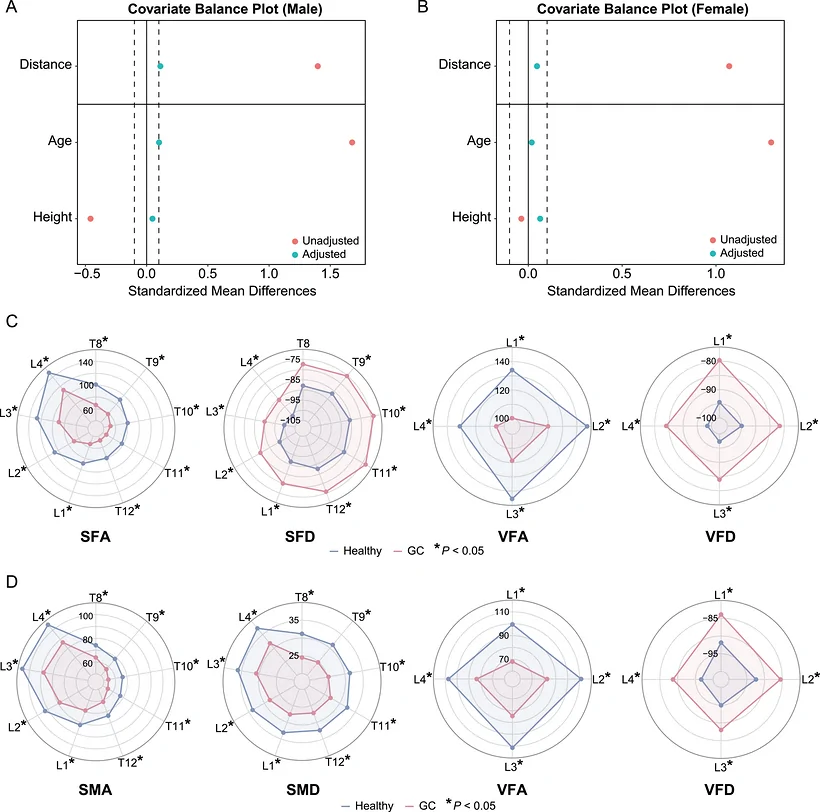
Propensity score matching (PSM) and comparison of body composition between GC patients and healthy controls. (A) Covariate balance plot for the male cohort, showing standardized mean differences of age and height before and after PSM. (B) Covariate balance plot for the female cohort. (C) Comparison of body composition parameters between healthy controls and GC patients in the matched male cohort. (D) Comparison of body composition parameters between healthy controls and GC patients in the matched female cohort.

Comparative analysis of body composition was performed between GC patients and health controls after PSM based on age and height (Figure 3A,B). Since most participants in the matched healthy cohort lacked L5 level coverage, differential analysis was conducted only for 2D parameters at vertebral levels from T8 to L4. Mean values of body composition parameters in both groups are visually summarized in radar charts, with asterisks denoting statistically significant differences determined by t‐tests. In males, compared to healthy controls, GC patients showed significantly lower SFA and subcutaneous fat index (SFI), along with significantly higher subcutaneous fat density (SFD) across all vertebral levels analyzed (Figure 3C, Figure S9). VFA and visceral fat index (VFI) were significantly reduced, while visceral fat density (VFD) was significantly increased. At specific vertebral levels, IMFA and intermuscular fat index (IMFI) were significantly decreased with increased intermuscular fat density (IMFD), whereas SMA, skeletal muscle index (SMI) and skeletal muscle density (SMD) showed no consistent significant differences (Figure S9). In females, GC patients demonstrated significantly lower SMA, SMI, SMD, VFA and VFI, alongside significantly higher VFD across all vertebral levels compared to healthy controls (Figure 3D, Figure S10). At specific vertebral levels, IMFA and IMFI were significantly reduced, while IMFD and subcutaneous fat parameters (SFA, SFI, SFD) showed no consistent significant alterations (Figure S10).

### 3.4 Prognostic Value of Multidimensional Body Composition in the Surgical Cohort

**FIGURE 4:**
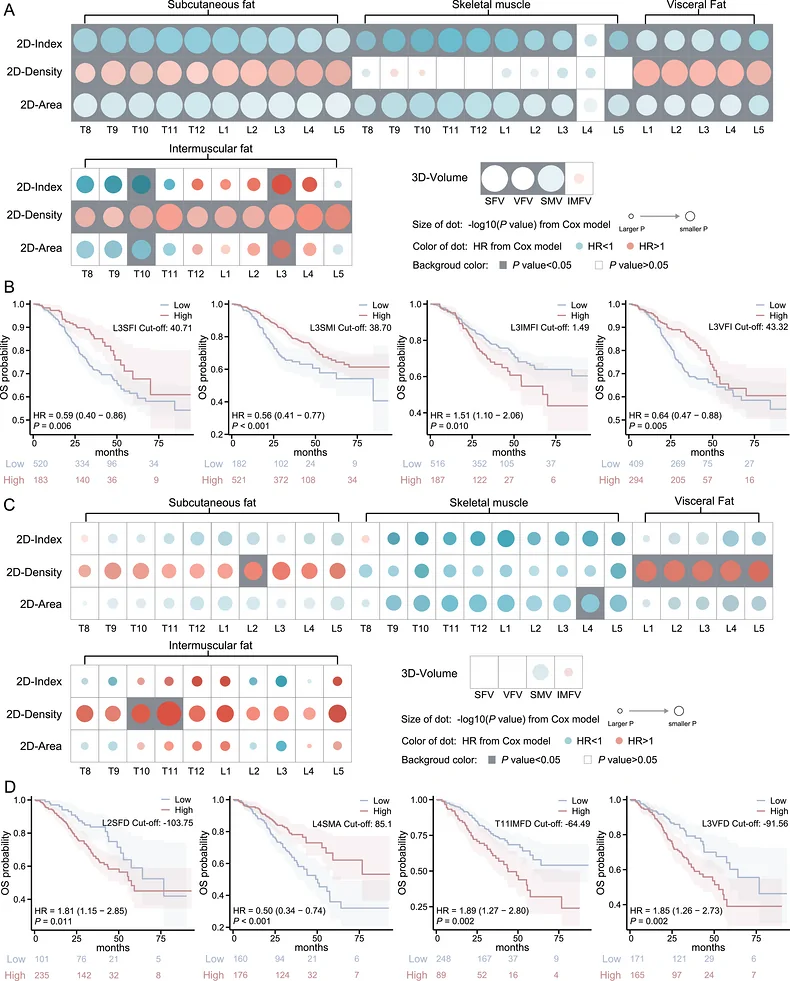
Prognostic value of multidimensional body composition parameters in the surgical cohort. (A) Heatmap of univariable Cox regression analysis for overall survival (OS) in males, showing Hazard Ratios (HR) for continuous body composition parameters. (B) Representative Kaplan–Meier survival curves for males at optimal cut‐off values of L3SMI, L3SFI, L3IMFI, and L3VFI. (C) Heatmap of univariable Cox regression analysis for OS in females. (D) Representative Kaplan–Meier survival curves for females at optimal cut‐off values of L2SFD, L4SMA, T11IMFD, and L3VFD. SFI, subcutaneous fat index; SFD, subcutaneous fat density; SMA, skeletal muscle area; SMI, skeletal muscle index; IMFI, intermuscular fat index; IMFD, intermuscular fat density; VFI, visceral fat index; VFD, visceral fat density.

Previous studies have reported an association between BMI and GC prognosis [22]. Similarly, we observed significantly longer OS in males with BMI > 20.7 and females with BMI > 22.03 in the surgical cohort (Figure S11A,B). Using the World Health Organization (WHO) BMI classification, we found that compared to the underweight group (BMI < 18.5), both the healthy weight (18.5 ≤ BMI < 25) and overweight/obesity (BMI ≥ 25) groups had significantly longer OS in males, with a similar trend observed in females (Figure S11C,D). A heatmap was generated based on the performance of continuous body composition parameters in univariable Cox regression analyses. In males, higher SFA, SFI, VFA, VFI, SMA, and SMI were associated with improved OS, while lower SFD, VFD, and IMFD correlated with better survival (Figure 4A). Optimal cutoff values for each body composition parameter were subsequently determined to maximize survival discrimination (Figure 4B, Figures S12–S16). Part of body composition parameters showed a similar association with OS in females (Figure 4C), with corresponding cutoff values determined (Figure 4D, Figure S17). Patients were subsequently stratified into high/low groups based on these cutoffs. To evaluate independent prognostic value, each body composition parameter was analyzed in a separate multivariable Cox regression model. Considering the substantial missingness in CEA (n = 192) and CA19‐9 (n = 326), as well as potential multicollinearity between body composition, age, and BMI, the primary multivariable models were adjusted for tumor stage, histopathological grade, and Her2 status. After correction for multiple hypothesis testing, 17 body composition parameters were significantly associated with OS in male patients, suggesting potential independent prognostic relevance (e.g., L1SMA: HR 0.438, ad *p* < 0.001; L3SMI: HR 0.513, ad *p* = 0.002; L5IMFD: HR 1.666, ad *p* = 0.020) (Table 2). Additional multivariable analyses incorporating age and BMI (Table S7), as well as CEA and CA19‐9 (Tables S8 and S9) were performed. Although statistical significance was attenuated after multiple testing correction in these extended models, the direction and magnitude of associations remained largely consistent. In females, after adjustment for tumor stage, histopathological grade and Her2 status, and correction for multiple testing, L4SMA remained independently associated with OS (HR 0.514, ad *p* = 0.003) (Table S10). Additional models incorporating age and BMI, as well as CEA and CA19‐9, yielded consistent trends (Tables S10 and S11), further supporting the robustness of these findings.

**TABLE 2:** Multivariable Cox Analyses of Body Composition in Male Surgery Cohorts.

**Parameters**	**HR (95%CI)**	***p* value**	**ad *p* value**	**Parameters**	**HR (95%CI)**	***p* value**	**ad *p* value**
T8SFA	0.696 (0.469 – 1.032)	0.072	0.109	L1IMFD	1.278 (0.912 – 1.789)	0.156	0.183
T8SFD	1.153 (0.780 – 1.705)	0.475	0.487	L2SFA	0.653 (0.466 – 0.916)	0.014*	0.052
T8SFI	0.746 (0.498 – 1.117)	0.155	0.183	L2SFD	1.539 (1.092 – 2.168)	0.014*	0.052
**T8SMA**	**0.556 (0.376 – 0.822)**	0.003*	0.016*	L2SFI	0.668 (0.476 – 0.940)	0.020*	0.052
T8SMI	0.645 (0.435 – 0.956)	0.029*	0.073	L2VFA	0.743 (0.524 – 1.053)	0.094	0.127
T8IMFD	0.938 (0.622 – 1.417)	0.763	0.763	**L2VFD**	**1.621 (1.122 – 2.341)**	0.010*	0.047*
T9SFA	0.648 (0.452 – 0.929)	0.018*	0.052	L2VFI	0.782 (0.555 – 1.104)	0.162	0.185
T9SFD	1.548 (1.080 – 2.217)	0.017*	0.052	**L2SMA**	**0.582 (0.417 – 0.813)**	0.001*	0.006*
T9SFI	0.636 (0.446 – 0.907)	0.012*	0.051	**L2SMI**	**0.552 (0.389 – 0.782)**	<0.001**	0.005*
**T9SMA**	**0.557 (0.389 – 0.799)**	0.001*	0.006*	L2IMFD	1.326 (0.940 – 1.871)	0.108	0.139
**T9SMI**	**0.506 (0.356 – 0.719)**	<0.001**	0.001*	L3SFA	0.563 (0.359 – 0.882)	0.012*	0.051
T9IMFD	1.154 (0.818 – 1.627)	0.414	0.430	L3SFD	1.417 (0.997 – 2.016)	0.052	0.090
T10SFA	0.731 (0.512 – 1.045)	0.086	0.121	L3SFI	0.620 (0.401 – 0.957)	0.031*	0.073
T10SFD	1.420 (1.002 – 2.012)	0.049*	0.087	L3VFA	0.709 (0.508 – 0.988)	0.043*	0.084
T10SFI	0.759 (0.532 – 1.083)	0.129	0.161	L3VFD	1.355 (0.956 – 1.922)	0.088	0.121
**T10SMA**	**0.499 (0.353 – 0.705)**	<0.001**	0.001*	L3VFI	0.655 (0.459 – 0.934)	0.019*	0.052
**T10SMI**	**0.515 (0.366 – 0.726)**	<0.001**	0.002*	**L3SMA**	**0.554 (0.392 – 0.781)**	<0.001**	<0.001**
T10IMFA	0.701 (0.502 – 0.979)	0.037*	0.078	**L3SMI**	**0.513 (0.360 – 0.730)**	<0.001**	0.002*
T10IMFD	1.512 (1.078 – 2.120)	0.017*	0.052	L3IMFA	1.521 (1.075 – 2.152)	0.018*	0.052
T10IMFI	0.704 (0.499 – 0.994)	0.046*	0.086	L3IMFD	1.326 (0.948 – 1.854)	0.100	0.133
T11SFA	0.683 (0.477 – 0.978)	0.037*	0.078	L3IMFI	0.657 (0.462 – 0.935)	0.019*	0.052
T11SFD	1.519 (1.077 – 2.144)	0.017*	0.052	L4SFA	0.660 (0.467 – 0.934)	0.019*	0.052
T11SFI	0.694 (0.483 – 0.997)	0.048*	0.087	L4SFD	1.365 (0.972 – 1.915)	0.072	0.109
**T11SMA**	**0.505 (0.359 – 0.709)**	<0.001**	0.001*	L4SFI	0.690 (0.485 – 0.982)	0.039*	0.078
**T11SMI**	**0.509 (0.361 – 0.717)**	<0.001**	0.001*	L4VFA	0.768 (0.542 – 1.087)	0.137	0.169
T11IMFD	1.418 (0.956 – 2.096)	0.080	0.114	L4VFD	0.663 (0.468 – 0.938)	0.020*	0.052
T12SFA	0.748 (0.521 – 1.073)	0.115	0.146	L4VFI	1.637 (0.220 – 12.195)	0.630	0.638
T12SFD	1.466 (1.028 – 2.092)	0.035*	0.078	L4IMFD	1.428 (1.019 – 2.001)	0.038*	0.078
T12SFI	0.694 (0.488 – 0.989)	0.044*	0.084	L5SFA	0.736 (0.523 – 1.037)	0.080	0.114
**T12SMA**	**0.519 (0.369 – 0.729)**	<0.001**	0.001*	L5SFD	1.474 (1.039 – 2.091)	0.030*	0.073
**T12SMI**	**0.552 (0.393 – 0.774)**	<0.001**	0.004*	L5SFI	0.769 (0.534 – 1.107)	0.158	0.183
T12IMFD	1.267 (0.891 – 1.802)	0.187	0.208	L5VFA	0.716 (0.500 – 1.028)	0.070	0.109
L1SFA	0.727 (0.509 – 1.037)	0.079	0.114	L5VFD	1.396 (0.986 – 1.974)	0.060	0.096
L1SFD	1.317 (0.945 – 1.837)	0.104	0.136	L5VFI	0.786 (0.539 – 1.145)	0.209	0.226
L1SFI	0.709 (0.498 – 1.010)	0.057	0.095	L5SMA	0.719 (0.510 – 1.014)	0.060	0.096
L1VFA	0.818 (0.574 – 1.166)	0.267	0.281	L5SMI	0.811 (0.562 – 1.170)	0.261	0.278
L1VFD	1.507 (1.033 – 2.198)	0.033*	0.075	**L5IMFD**	**1.666 (1.181 – 2.350)**	0.004*	0.020*
L1VFI	0.794 (0.562 – 1.121)	0.190	0.208	SFV	0.709 (0.501 – 1.004)	0.053	0.090
**L1SMA**	**0.438 (0.312 – 0.616)**	<0.001**	0.001*	VFV	0.789 (0.562 – 1.107)	0.170	0.192
**L1SMI**	**0.491 (0.350 – 0.689)**	<0.001**	0.001*	SMV	0.779 (0.557 – 1.092)	0.147	0.178

*Note*. Each body composition parameter was analyzed separately in an individual multivariable Cox regression model. HR was adjusted with Grade, Stage and Her2 (Low group as reference).

### 3.5 Association between Multidimensional Body Composition and Patient Survival in the ICI Cohort

Similarly, a heatmap was generated based on the performance of various body composition metrics in univariable Cox regression analyses. In female patients, higher SFA and SFI were associated with improved OS, while lower SFD and IMFD were correlated with better OS (Figures S18B and S19). Male patients exhibited similar trends in the impact of body composition parameters on OS (Figure S18A). Multivariable Cox regression analyses were conducted following the similar analytical framework as described for the surgical cohort. After adjustment for clinical stage, histopathological grade, Her2 status and PD‐L1 expression, and correction for multiple hypothesis testing, 5 body composition parameters remained significantly associated with mortality in female patients (e.g., L3SFA: HR 0.957, ad *p* value = 0.023; L3SFI: HR 0.889, ad *p* value = 0.023) (Table S12). Additional multivariable models incorporating age and BMI (Table S12), as well as models adjusted for CEA and CA19‐9 (Table S13) were performed.

### 3.6 Exploratory Analysis of the Association of Multidimensional Body Composition With Tumor Pathological and Molecular Features

**FIGURE 5:**
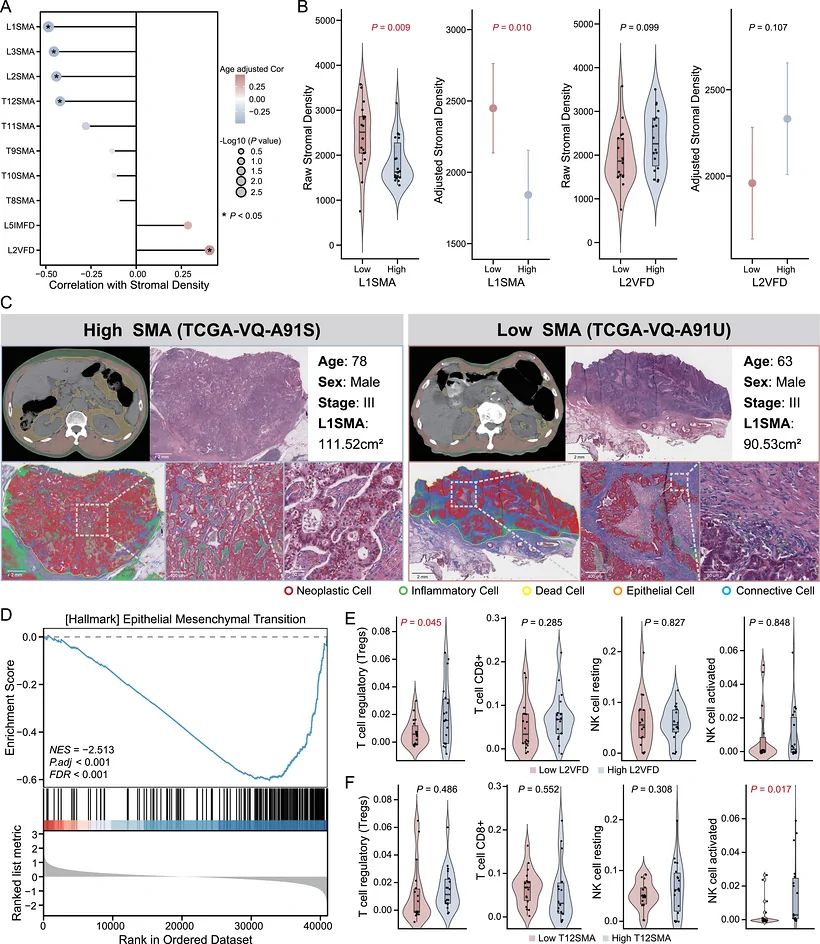
Association of body composition with pathological features and transcriptomic profiles. (A) Age‐adjusted Spearman correlation analysis between body composition parameters and stromal density. (B) Comparison of stromal density between groups stratified by L1SMA and L2VFD, showing the raw data distribution as well as age‐adjusted means with 95% confidence intervals. (C) Representative pathological sections from high‐L1SMA and low‐L1SMA patients. (D) GSEA showing the negative association between L1SMA and the Hallmark “Epithelial Mesenchymal Transition” pathway. (E) Immune infiltration analysis using the CIBERSORT algorithm comparing high and low L2VFD groups, showing significantly higher Tregs infiltration in the high L2VFD group. (F) Immune infiltration analysis comparing high and low T12SMA groups, showing significantly higher NK cell activated infiltration in the high T12SMA group. SMA, skeletal muscle area; VFD, visceral fat density.

Leveraging the TCGA‐STAD cohort, we investigated the biological underpinnings through both pathological and transcriptomic analyses. Due to the limited number of eligible female patients (n = 7), this section focuses exclusively on male data. At the pathological level, we analyzed 39 male patients with matched CT images and pathological data. Spearman correlation analysis revealed that T12SMA, L1SMA, L2SMA and L3SMA were significantly negatively correlated with stromal density, while L2VFD showed a positive correlation (Figure 5A). Significant differences in stromal density were observed between the high and low SMA groups with age‐adjusted (Figure 5B). Representative pathological slides with cell classification results from one patient each in the high and low SMA groups were shown in Figure 5C. We analyzed transcriptomic data from 36 male patients with matched CT images and RNA‐seq data. GSEA demonstrated that higher L1SMA and L2SMA were significantly associated with inhibition of the “Epithelial Mesenchymal Transition” (EMT) pathway (FDR < 0.001). Elevated L1SMA was linked to suppression of the “ECM Receptor Interaction” pathway (FDR < 0.001) (Figure 5D, Figure S21A,B). Furthermore, increased SMA and decreased VFD were correlated with the upregulation of the “Neuroactive Ligand Receptor Interaction” pathway (all FDR < 0.001) (Figure S21B–E). Immune infiltration analysis was performed using the CIBERSORT (Figure 5E,F, Figure S22) and MCPCOUNTER (Figure S23) algorithms, with age adjustment applied for intergroup comparisons. The results showed increased T cell regulatory (Tregs) infiltration in the high VFD group and increased activated NK cell infiltration in the high SMA group.

### 3.7 Construction of Prognostic Prediction Models

**FIGURE 6:**
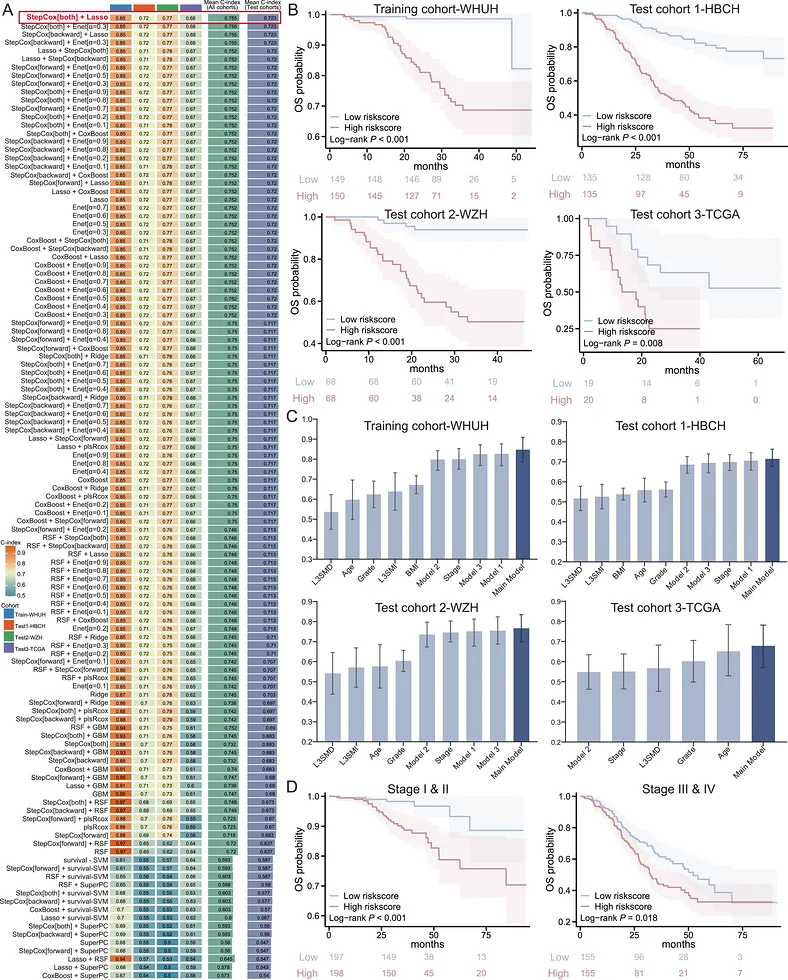
Prognostic prediction model for male patients. (A) Average C‐index across training and test cohorts for 117 models, “Stepcox[both] + Lasso” was ultimately selected as the main model for subsequent analyses. (B) Kaplan‐Meier curves stratified by risk group in training and test cohorts. (C) Comparison of C‐indices among the main model, other models and clinical variables. Model 1: clinical variables + L3SMI; Model 2: clinical variables + L3SMD; Model 2: clinical variables + L3SMI + L3SMD. (D) Risk stratification Kaplan‐Meier curves for stage I–II (left) and stage III–IV (right) patients.

We constructed sex‐specific prognostic prediction models integrating clinical variables with multidimensional body composition features. In male GC patients, among 117 candidate models, the StepCox[both] + Lasso model demonstrated the best overall performance and was selected as the main model, achieving the highest average C‐index (0.723) across external test datasets (Figure 6A). Male patients were divided into high‐risk and low‐risk groups based on the median risk score, with the high‐risk group showing significantly lower OS (all *p* < 0.05) (Figure 6B). Compared with models based solely on clinical variables or traditional body composition features (L3SMI and L3SMD), the main model consistently achieved higher predictive performance (Figure 6C) [23, 24, 25]. Specifically, its performance exceeded that of models incorporating single clinical factors, traditional body composition features, as well as combined models integrating clinical variables with L3SMI, L3SMD, or both. Notably, the model further refined prognostic stratification within clinical stage subgroups in the surgical cohorts, with significant survival differences observed in both stage I‐II (*p* < 0.001) and stage III‐IV patients (*p* = 0.018) (Figure 6D). In females, the same methodology was applied for model construction. The results indicated that the stepCox[forward] model achieved the highest average C‐index (0.705) in the test sets (Figure S24A). This model also yielded the highest C‐index in both the training and test sets, and patients in the high‐risk group similarly exhibited significantly lower OS compared to those in the low‐risk group (Figure S24B). In stage stratified analyses, the model retained prognostic discrimination in advanced‐stage patients (stage III‐IV, *p* = 0.003), while the difference was less pronounced in early‐stage patients (stage I‐II, *p* = 0.062), likely reflecting the limited sample size in this subgroup (Figure S24C). Decision curve analysis demonstrates that the models exhibited favorable clinical applicability in both male and female patients (Figure S25).

## 4 Discussion

Based on a multicenter retrospective cohort comprising 1196 gastric cancer patients and 983 healthy controls from three medical centers across China, this study employed a pre‐trained nnU‐Net model to perform fully automated segmentation of subcutaneous fat, skeletal muscle, intermuscular fat and visceral fat from abdominal CT images. We systematically evaluated the prognostic value of these body composition parameters across different treatment modalities, constructed and validated sex‐specific prognostic models integrating body composition with clinical variables, and conducted preliminary exploration of their underlying biological mechanisms at the tumor microenvironment and molecular levels.

One notable findings was the identification of distinct body composition alterations in GC patients compared with healthy controls after PSM, with evident sex‐specific patterns. Male patients primarily exhibited reductions in subcutaneous and visceral fat, whereas female patients showed predominant reductions in skeletal muscle and visceral fat. The androgen milieu or sex‐specific endocrine functions of fat might render it more susceptible to being utilized as a “fuel depot” for tumor metabolism in male patients [26]. In contrast, the greater skeletal muscle loss observed in females may be related to lower baseline muscle reserves and increased susceptibility to tumor‐associated catabolic processes. These observations suggest that sex‐specific factors should be considered when evaluating the nutritional and metabolic status of GC patients, and highlight the potential value of detailed body composition assessment.

Regarding prognostic value, this study further confirms that multidimensional body composition is significantly associated with OS, independent of clinical stage, histopathological grade, and conventional tumor markers. In the surgical cohort, higher muscle and fat areas/indices and lower fat density were associated with longer OS. Notably, in male patients, several parameters remained significantly associated with OS after adjusting for confounders. This aligns with previous reports linking sarcopenia and fatty infiltration to poor prognosis [25, 27]. Low muscle mass may directly contribute to increased postoperative complications [28, 29]. Elevated fat density, potentially resulting from inflammatory cell infiltration and fibrosis within fat, signifies a systemic inflammatory state and fat dysfunction. This “dysfunctional fat” phenotype might promote tumor progression via the secretion of abundant pro‐inflammatory factors [30, 31]. Notably, in the ICI cohort, survival benefit in female patients was associated with higher SFA, which is consistent with the “obesity paradox” in the context of ICI therapy [32]. One possible explanation is that adipose tissue may influence systemic inflammatory and immune status, thereby modulating the tumor immune microenvironment and potentially enhancing responsiveness to ICI therapy [32, 33].

To further explore potential biological underpinnings, we integrated pathological and transcriptomic data from the TCGA‐STAD cohort. In male GC patients, higher SMA was associated with lower tumor stromal density, whereas higher VFD showed the opposite trend, and these associations remained consistent after age adjustment. At the transcriptomic level, higher SMA was linked to downregulation of pathways related to EMT and ECM interactions, as well as the upregulation in neuroactive ligand‐receptor signaling. In addition, immune deconvolution analyses suggested differential immune infiltration patterns, including increased activated NK cell infiltration in patients with higher SMA and increased Tregs infiltration in those with higher VFD. Taken together, these findings suggest that multidimensional body composition may be associated with tumor microenvironmental features, including stromal composition, extracellular matrix remodeling, and immune regulation. Existing research suggests multifaceted interactions between tumor stromal fibroblasts and tumor cells contribute to poor patient outcomes. For instance, stromal fibroblasts can produce aspartate and glutamate to promote tumor metabolism [34], secrete specific factors to recruit and activate macrophages, establishing key pathways driving immunosuppression and tumor progression [35, 36], and enhance the tumor EMT pathway by modulating miRNA activity, thereby stimulating GC cell migration and invasion [37, 38]. This provide a possible explanation is that systemic metabolic and inflammatory states reflected by muscle and adipose tissue may influence tumor‐stroma interactions and immune cell dynamics. However, given the limited sample size and the observational nature of these analyses, these findings should be interpreted as exploratory and hypothesis‐generating, rather than indicative of causal mechanisms. Further studies are warranted to validate these associations and clarify their biological relevance.

Building upon these prognostic insights, we developed sex‐specific prognostic models that integrate multidimensional body composition with clinical variables. In male patients, the StepCox[both]+Lasso model demonstrated the most stable performance across cohorts, achieving an average C‐index of 0.723 in external test datasets. In females, a forward stepwise Cox model showed comparable performance, with an average C‐index of 0.705. These models consistently stratified patients into distinct risk groups with significantly different survival outcomes, and further refined prognostic discrimination within clinical stage subgroups in the surgical cohorts. Previous research has primarily concentrated on the prognostic value of L3‐level muscle characteristics in patients [24, 25, 39]. Notably, the integrated models outperformed those based on clinical variables alone or conventional body composition metrics such as L3SMI and L3SMD, suggesting that incorporating multidimensional features may provide additional prognostic information. From a clinical perspective, these findings indicate that body composition‐derived features may complement traditional risk factors for individualized prognostic assessment. However, the performance differences across sex and disease stages, as well as the moderate sample size in certain subgroups, suggest that these models should be interpreted cautiously. Further prospective validation and evaluation in diverse populations are warranted to confirm their clinical utility.

This study extends existing research on body composition in GC by incorporating several methodological and analytical strengths. First, the application of an AI‐based automated framework enabled multi‐level and multidimensional quantification of abdominal CT images, providing a more comprehensive and reproducible assessment of body composition compared with conventional manual or single‐slice approaches [40, 41]. Second, by leveraging large multicenter GC cohorts together with a health screening population, we systematically compared body composition differences between patients and healthy individuals using PSM, thereby revealing sex‐specific patterns of body composition alteration in GC and their profound association with prognosis. The consistency of findings was validated across multiple treatment cohorts. In contrast, existing studies often rely on single‐center, small‐sample data [42]. Third, beyond association analyses, we developed and externally validated prognostic models integrating body composition with clinical variables, which demonstrated improved risk stratification compared with models based on conventional indicators alone. In addition, this study incorporated histopathological and transcriptomic data to explore potential links between systemic body composition and tumor microenvironmental features. While these analyses remain exploratory, they provide complementary evidence supporting the potential biological relevance of body composition from multiple perspectives, including stromal characteristics, EMT‐related pathways, and immune cell infiltration. Overall, these findings contribute to a more comprehensive understanding of body composition in GC and suggest its potential role as a complementary biomarker for prognosis and risk stratification, while also providing a basis for future investigations into underlying mechanisms and potential clinical applications.

This study has several limitations. First, despite involving multicenter cohorts, this retrospective study might still be subject to selection and information biases, and the sex imbalance in sample size might reduce the statistical power in the female subgroup. Second, tumor microenvironment analyses were conducted in a relatively small subset of patients and were limited to correlative observations without functional validation. Although strong associations were observed, causal relationships between body composition and tumor biology warrant further prospective and mechanistic studies. Finally, the clinical applicability of the prognostic models requires further validation in prospective studies before routine implementation.

## 5 Conclusion

This multicenter study leveraged deep learning to systematically analyze multidimensional body composition from CT images in GC cohorts. Our findings suggest that GC is associated with distinct, sex‐specific alterations in body composition, and that skeletal muscle and adipose tissue–related parameters are significantly associated with OS. Integrative analyses incorporating pathological and transcriptomic data further indicated potential links between body composition and tumor microenvironmental features, including EMT‐related pathways and immune cell infiltration. Furthermore, prognostic models integrating multidimensional body composition with clinical variables demonstrated consistent discriminatory performance across independent cohorts, suggesting their potential utility for risk stratification. This study provides a comprehensive, multi‐level perspective linking systemic physiological status with clinical outcomes in GC, and highlights body composition as a potential complementary biomarker for prognosis. Further prospective and mechanistic studies are warranted to validate these findings and clarify their clinical and biological implications.

## Author Contributions

T.X.L., T.T.L., and Q.Y. conceived and designed the study, and were involved in data interpretation. T.X.L., T.T.L., Q.Y., Z.Q.Z., X.F.G., X.W., Y.H.Z., L.M.G., Y.S., W.J.W., W.H.G., and Z.W. participated in data acquisition and analysis. T.X.L. and T.T.L. drafted the initial manuscript. L.Y. and Y.F.N. jointly supervised the project and critically revised the manuscript for intellectual content.

## Ethics Approval and Consent to Participate

This Study Was Approved By the Ethics Committees of Union Hospital, Tongji Medical College, Huazhong University of Science and Technology (2025‐0852), Hubei Cancer Hospital (LLHBCH2025YN‐067), and the First Affiliated Hospital of Wenzhou Medical University (KY2022‐202). All patient Information Was Anonymized, and written informed consent was waived due to the retrospective nature of the study.

## Conflicts of Interest

The authors declare no conflicts of interest.

## Supporting information




**Supporting File**: advs75859‐sup‐0001‐SuppMat.docx.

## Data Availability

Data will be available upon reasonable request to the corresponding author (yanfeng_niu@hust.edu.cn).
