# Supplementary material for: Artificial Intelligence‐Based Body Composition Analysis Reveals Sex‐Specific Prognostic Markers and Their Clinical Value in Gastric Cancer: A Multicenter Study
Source: Adv Sci (Weinh). 2026 Jun 1;13(47):e75859. doi: 10.1002/advs.75859 (PMC13335994; doi:10.1002/advs.75859)
Supplement: Supplementary file 1 [file ADVS-13-e75859-s001.docx]

**Supplementary Material**

**Artificial Intelligence-Based Body Composition Analysis Reveals Sex-Specific Prognostic Markers and Their Clinical Value in Gastric Cancer: A Multicenter Study.**

**Table of Contents**

**[Supplementary Material	1](#_Toc18687)**

**[Supplementary Figures and Figure legends	4](#_Toc27215)**

[Figure S1. Rationale for analysis level selection in body composition assessment.	4](#_Toc22357)

[Figure S2. Assessment of the segmentation algorithm's reliability.	5](#_Toc27405)

[Figure S3. Distribution of body composition volumes across four gastric cancer cohorts.	6](#_Toc30331)

[Figure S4. Correlations of body composition volumes with clinical variables.	7](#_Toc15266)

[Figure S5. Correlation analysis of different body composition parameters at the same anatomical level in male patients.	8](#_Toc17483)

[Figure S6. Correlation analysis of different body composition parameters at the same anatomical level in female patients.	9](#_Toc13991)

[Figure S7. Correlation analysis of the same body composition parameter across different levels in male patients.	10](#_Toc15484)

[Figure S8. Correlation analysis of the same body composition parameter across different levels in female patients.	11](#_Toc20081)

[Figure S9. Comparison of body composition between male GC patients and healthy controls.	12](#_Toc16291)

[Figure S10. Comparison of body composition between female GC patients and healthy controls.	13](#_Toc654)

[Figure S11. KM survival curves showing the relationship between BMI and OS in GC patients of the surgical cohort.	14](#_Toc4917)

[Figure S12. Relationship of T8, T9 and T10 level body compositions with OS in male GC patients of the surgical cohort.	15](#_Toc15927)

[Figure S13. Relationship of T11 and T12 level body compositions with OS in male GC patients of the surgical cohort.	16](#_Toc10941)

[Figure S14. Relationship of L1 and L2 level body compositions with OS in male GC patients of the surgical cohort.	17](#_Toc20276)

[Figure S15. Association between partial L3 level body compositions and OS in male GC patients of the surgical cohort.	18](#_Toc19364)

[Figure S16. Relationship of L4 and L5 level body compositions with OS in male GC patients of the surgical cohort.	19](#_Toc24841)

[Figure S17. Association between specific multidimensional body composition and OS in female GC patients of the surgical cohort.	20](#_Toc26673)

[Figure S18. Prognostic value of multidimensional body composition parameters in the ICI cohort.	21](#_Toc1635)

[Figure S19. Association between specific multidimensional body composition and OS in female GC patients of the ICI cohort.	22](#_Toc13241)

[Figure S20. Correlation and difference analyses of body composition and pathological parameters with age adjustment.	23](#_Toc27427)

[Figure S21. Gene set enrichment analysis (GSEA) based on body composition stratification with age adjustment.	24](#_Toc16657)

[Figure S22. Immune infiltration analysis was performed using the CIBERSORT algorithm with age adjustment.	25](#_Toc23412)

[Figure S23. Immune infiltration analysis was performed using the MCPCOUNTER algorithm with age adjustment.	26](#_Toc17850)

[Figure S24. Prognostic prediction model for female patients.	27](#_Toc28872)

[Figure S25. Decision curve analysis of the models for predicting 2-year OS in both male and female GC patients.	28](#_Toc12142)

**[Supplementary Tables	29](#_Toc31681)**

[Table S1. Quantification and definitions of multidimensional body composition parameters	29](#_Toc26342)

[Table S2. Definition and computational approaches for spatial pathological parameters	30](#_Toc14921)

[Table S3. Features incorporated during model construction	31](#_Toc12829)

[Table S4. Baseline characteristics of healthy controls	31](#_Toc9053)

[Table S5. Baseline characteristics of study participants after PSM (Male GC patients and healthy controls, matched for age and height)	32](#_Toc31065)

[Table S6. Baseline characteristics of study participants after PSM (Female GC patients and healthy controls, matched for age and height)	32](#_Toc7779)

[Table S7. Multivariable cox analyses of body composition in male surgery cohorts (adjusted for age, BMI, Grade, Stage, and Her2)	33](#_Toc3514)

[Table S8. Multivariable cox analyses of body composition in male surgery cohorts (adjusted for CEA, CA199, Grade, Stage, and Her2)	34](#_Toc27138)

[Table S9. Multivariable cox analyses of body composition in male surgery cohorts (adjusted for age, BMI, CEA, CA199, Grade, Stage, and Her2)	35](#_Toc17711)

[Table S10. Multivariable cox analyses of body composition in female surgery cohorts (adjusted for age, BMI, Grade, Stage, Her2)	36](#_Toc22221)

[Table S11. Multivariable cox analyses of body composition in female surgery cohorts (adjusted for age, BMI, CEA, CA199, Grade, Stage, Her2)	36](#_Toc7918)

[Table S12. Multivariable cox analyses of body composition in female ICI cohorts (adjusted for age, BMI, Grade, Stage, Her2, PDL1 CPS)	37](#_Toc23626)

[Table S13. Multivariable cox analyses of body composition in female ICI cohorts (adjusted for age, BMI, CEA, CA199, Grade, Stage, Her2, PDL1 CPS)	38](#_Toc31980)

## Supplementary Figures and Figure legends

## Figure S1. Rationale for analysis level selection in body composition assessment.

**Figure S1.** Due to the susceptibility of the T7 level and above to artifacts from arm positioning and inconsistent scan coverage, body composition analysis was restricted to the T8 level and below in this study.

## Figure S2. Assessment of the segmentation algorithm's reliability.

**Figure S2.** Bland-Altman plots show excellent agreement (ICC > 0.89) between the AI algorithm and manual measurements by a radiologist for (A) subcutaneous fat area (SFA), (B) visceral fat area (VFA), (C) skeletal muscle area (SMA), and (D) intermuscular fat area (IMFA) at the L3 level in 50 randomly selected patients.

## Figure S**3. Distribution of body composition volumes across four gastric cancer cohorts.**

**Figure S3.** Distributions of (A) Subcutaneous fat volume (SFV), (B) Visceral fat volume (VFV), (C) Skeletal muscle volume (SMV), and (D) Intermuscular fat volume (IMFV) among the four independent gastric cancer cohorts.

## Figure S4. Correlations of body composition volumes with clinical variables.

**Figure S4**. Correlation analyses of SFV /VFV/IMFV with BMI (A) and Age (B).

## Figure S5. Correlation analysis of different body composition parameters at the same anatomical level in male patients.

**Figure S5.** Correlation analysis of different body composition parameters at the same anatomical level in male patients.

## Figure S6. Correlation analysis of different body composition parameters at the same anatomical level in female patients.

**Figure S6.** Correlation analysis of different body composition parameters at the same anatomical level in female patients.

## Figure S7. Correlation analysis of the same body composition parameter across different levels in male patients.

**Figure S7.** Correlation analysis of the same body composition parameter across different levels in male patients.

## Figure S8. Correlation analysis of the same body composition parameter across different levels in female patients.

**Figure S8.** Correlation analysis of the same body composition parameter across different levels in female patients.

## Figure S9. Comparison of body composition between male GC patients and healthy controls.

**Figure S9.** Comparison of body composition between male GC patients and healthy controls.

## Figure S10. Comparison of body composition between female GC patients and healthy controls.

**Figure S10.** Comparison of body composition between female GC patients and healthy controls.

## Figure S11. KM survival curves showing the relationship between BMI and OS in GC patients of the surgical cohort.

**Figure S11.** Male (A) and female (B) patients were grouped based on the cutoff value which determined using the surv_cutpoint function with the minimum p-value. Male (C) and female (D) patients were grouped based on the standard WHO BMI classification.

## Figure S12. Relationship of T8, T9 and T10 level body compositions with OS in male GC patients of the surgical cohort.

**Figure S12.** KM survival curves showing the relationship of T8, T9 and T10 level body compositions with OS in male GC patients of the surgical cohort.

## Figure S13. Relationship of T11 and T12 level body compositions with OS in male GC patients of the surgical cohort.

**Figure S13.** KM survival curves showing the relationship of T11 and T12 level body compositions with OS in male GC patients of the surgical cohort.

## Figure S14. Relationship of L1 and L2 level body compositions with OS in male GC patients of the surgical cohort.

**Figure S14.** KM survival curves showing the relationship of L1 (A) and L2 (B) level body compositions with OS in male GC patients of the surgical cohort.

## Figure S15. Association betwe**en partial L3 level bod**y compositions and OS in male GC patients of the surgical cohort.

**Figure S15.** KM survival curves showing the relationship of partial L3 level body compositions with OS in male GC patients of the surgical cohort.

## Figure S16. Relationship of L4 and L5 level body compositions with OS in male GC patients of the surgical cohort.

**Figure S16.** KM survival curves showing the relationship of L4 (A) and L5 (B) level body compositions with OS in male GC patients of the surgical cohort.

## Figure S17. Association between specific multidimensional body composition and OS in female GC patients of the surgical cohort.

**Figure S17.** Association between specific multidimensional body composition and OS in female GC patients of the surgical cohort.

## Figure S18. Prognostic value of multidimensional body composition parameters in the ICI cohort.

**Figure S18.** (A) Heatmap of univariable cox regression analysis for overall survival (OS) in males. (B) Heatmap of univariable cox regression analysis for OS in females.

## Figure S19. Association between specific multidimensional body composition and OS in female GC patients of the ICI cohort.

**Figure S19.** Association between specific multidimensional body composition and OS in female GC patients of the ICI cohort.

## Figure S20. Correlation and difference analyses of body composition and pathological parameters with age adjustment.

**Figure S20.** (A) Associations between body composition with Tumor-Immune Colocalization, Average Tumor-Inflammatory Distance, Ripley’s K and Inflammatory Density. (B) Comparison of Stromal density between high and low SMA groups.

## Figure S21. Gene set enrichment analysis (GSEA) based on body composition stratification with age adjustment.

**Figure S21.** Using the “low group” as reference, gene expression data were adjusted for age prior to GSEA. (A) L1SMA; (B) L2SMA; (C) T12SMA; (D) L3SMA; (E) L2VFD. Enrichment plots illustrate representative pathways associated with differences in body composition. NES, normalized enrichment score; FDR, false discovery rate.

## Figure S22. **Immune infiltration analysis was performed using the CIBERSORT algorithm** with age adjustment**.**

**Figure S22.** Immune infiltration analysis was performed using the CIBERSORT algorithm to compare the high/low L2VFD groups (A), T12SMA groups (B), L1SMA group (C), L2SMA group (D), and L3SMA group (E).

## Figure S23. **Immune infiltration analysis was performed using the MCPCOUNTER algorithm** with age adjustment**.**

**Figure S23.** Immune infiltration analysis was performed using the MCPCOUNTER algorithm to compare the high/low T12SMA groups (A), L1SMA group (B), L2SMA group (C), L3SMA group (D), and L2VFD groups (E).

## Figure S24. Prognostic prediction model for female patients.

**Figure S24.** (A) Average C-index across training and test cohorts for 117 models, “Stepcox[forward]” was ultimately selected as the main model for subsequent analyses. (B) Kaplan-Meier curves stratified by risk group in training and test cohorts (left), and comparison of C-indices among the main model, other models and clinical variables (right). Model 1: clinical variables + L3SMI; Model 2: clinical variables + L3SMD; Model 2: clinical variables + L3SMI + L3SMD. (C) Risk stratification Kaplan-Meier curves for stage I–II (left) and stage III–IV (right) patients.

## Figure S25. Decision curve analysis of the models for predicting 2-year OS in both male and female GC patients.

**Figure S25.** Decision curve analysis of the models for predicting 2-year OS male (A) and female (B) GC patients in training and test cohorts.

## Supplementary Tables

## Table S1. Quantification and definitions of multidimensional body composition parameters

| **Category** | **Parameters** | **Full name** | **Range** | **Calculation** |
| --- | --- | --- | --- | --- |
| 3D-Volume | SFV | Subcutaneous fat volume | L1-L5 | Calculated as voxel volume × number of segmented voxels within the L1-L5 region (cm^3^). |
|  | SMV | Skeletal muscle volume |  |  |
|  | IMFV | Intermuscular fat volume |  |  |
|  | VFV | Visceral fat volume |  |  |
| 2D-Area | SFA | Subcutaneous fat area | T8, T9, T10, T11, T12, L1, L2, L3, L4, L5 | Determine the superior and inferior boundary information of each vertebral space along the Z-axis, and calculate the cross-sectional area of the body composition mask at the mid-vertebral slice (cm^2^). |
|  | SMA | Skeletal muscle area |  |  |
|  | IMFA | Intermuscular fat area |  |  |
|  | VFA | Visceral fat area | L1, L2, L3, L4, L5 |  |
| 2D-Density | SFD | Subcutaneous fat density | T8, T9, T10, T11, T12, L1, L2, L3, L4, L5 | Pair the original images with the aforementioned 2D body composition mask to extract mean CT attenuation (Hounsfield units, HU) for each body composition. |
|  | SMD | Skeletal muscle density |  |  |
|  | IMFD | Intermuscular fat density |  |  |
|  | VFD | Visceral fat density | L1, L2, L3, L4, L5 |  |
| 2D-Index | SFI | Subcutaneous fat index | T8, T9, T10, T11, T12, L1, L2, L3, L4, L5 | Calculated as cross-sectional area normalized by height squared (cm^2^/m^2^). |
|  | SMI | Skeletal muscle index |  |  |
|  | IMFI | Intermuscular fat index |  |  |
|  | VFI | Visceral fat index | L1, L2, L3, L4, L5 |  |

## Table S2. Definition and computational approaches for spatial pathological parameters

| **Category** | **Metric** | **Definition** | **Calculation Method** | **Unit** |
| --- | --- | --- | --- | --- |
| Basic Parameters | Tumor Area | Total annotated tumor region area | Sum of all tumor-annotated regions × pixel calibration | mm² |
| Cellular Composition | Inflammatory Cells | Immune cells within tumor microenvironment | Count of “Inflammatory” classified cells | cells |
|  | Connective Cells | Stromal/connective tissue cells | Count of “Connective” classified cells | cells |
| Cellular Density | Immune Infiltration | Inflammatory cell density | Inflammatory count / Tumor Area | cells/mm² |
|  | Stromal Density | Connective cell density | Connective count / Tumor Area | cells/mm² |
| Spatial Distribution | Average Tumor-Inflammatory Distance | Mean minimal distance between tumor and immune cells | For each tumor cell, find nearest inflammatory cell using gridded search; average all distances | µm |
|  | Tumor-Immune Colocalization | Proportion of tumor cells in close proximity to immune cells | Tumor cells within 50µm of any inflammatory cell / total tumor cells | ratio |
|  | Ripley’ s K | Spatial clustering index of inflammatory cells | For each inflammatory cell, count neighbors within 100µm radius using optimized grid search; normalize by expected count under complete spatial randomness: K(r) = [Σcount / (n × λ)] / (πr²) | / |

## Table S3. Features incorporated during model construction

| **Features** | | | | | |
| --- | --- | --- | --- | --- | --- |
| Age | Grade | Stage | T8SFA | T8SFD | T8SMA |
| T8SMD | T8IMFA | T8IMFD | T9SFA | T9SFD | T9SMA |
| T9SMD | T9IMFA | T9IMFD | T10SFA | T10SFD | T10SMA |
| T10SMD | T10IMFA | T10IMFD | T11SFA | T11SFD | T11SMA |
| T11SMD | T11IMFA | T11IMFD | T12SFA | T12SFD | T12SMA |
| T12SMD | T12IMFA | T12IMFD | L1SFA | L1SFD | L1VFA |
| L1VFD | L1SMA | L1SMD | L1IMFA | L1IMFD | L2SFA |
| L2SFD | L2VFA | L2VFD | L2SMA | L2SMD | L2IMFA |
| L2IMFD | L3SFA | L3SFD | L3VFA | L3VFD | L3SMA |
| L3SMD | L3IMFA | L3IMFD | L4SFA | L4SFD | L4VFA |
| L4VFD | L4SMA | L4SMD | L4IMFA | L4IMFD | L5SFA |
| L5SFD | L5VFA | L5VFD | L5SMA | L5SMD | L5IMFA |
| L5IMFD | SFV | VFV | SMV | IMFV |  |

## Table S4. Baseline characteristics of healthy controls

| **Characteristics** | **Healthy** | **GC** | ***P* value** |
| --- | --- | --- | --- |
| n | 1959 | 1339 |  |
| age, median (IQR) | 41 (34, 56) | 40 (33, 52) | 0.002 |
| height, median (IQR) | 1.72 (1.68, 1.75) | 1.59 (1.56, 1.63) | < 0.001 |
| weight, median (IQR) | 74 (67, 80.80) | 57 (51.83, 62) | < 0.001 |
| BMI, median (IQR) | 25.28 (23.26, 27.11) | 22.27 (20.60, 24.39) | < 0.001 |

Note. BMI: Body mass index

## Table S5. Baseline characteristics of study participants after PSM (Male GC patients and healthy controls, matched for age and height)

| **Characteristics** | **Healthy** | **GC** | ***P* value** |
| --- | --- | --- | --- |
| n | 639 | 639 |  |
| age, median (IQR) | 59 (52, 64) | 59 (54, 65) | 0.037 |
| height, median (IQR) | 1.69 (1.65, 1.73) | 1.7 (1.65, 1.73) | 0.235 |
| weight, median (IQR) | 71 (65, 78.40) | 65 (58, 71) | < 0.001 |
| BMI, median (IQR) | 25.19 (23.19, 26.84) | 22.49 (20.57, 24.62) | < 0.001 |

Note. BMI: Body mass index

## Table S6. Baseline characteristics of study participants after PSM (Female GC patients and healthy controls, matched for age and height)

| **Characteristics** | **Healthy** | **GC** | ***P* value** |
| --- | --- | --- | --- |
| n | 344 | 344 |  |
| age, median (IQR) | 58 (49, 63) | 56.5 (50, 65) | 0.966 |
| height, median (IQR) | 1.59 (1.55, 1.63) | 1.60 (1.55, 1.62) | 0.406 |
| weight, median (IQR) | 58 (52.98, 63.05) | 55 (50, 61) | < 0.001 |
| BMI, median (IQR) | 23.02 (21.16, 24.87) | 21.52 (19.81, 24.22) | < 0.001 |

Note. BMI: Body mass index

## Table S7. Multivariable cox analyses of body composition in male surgery cohorts (adjusted for age, BMI, Grade, Stage, and Her2)

| **Parameters** | **HR (95%CI)** | ***P* value** | **ad *P* value** | **Parameters** | **HR (95%CI)** | ***P* value** | **ad *P* value** |
| --- | --- | --- | --- | --- | --- | --- | --- |
| T8SFA | 0.789 (0.487 - 1.279) | 0.336 | 0.637 | L1IMFD | 1.125 (0.796 - 1.590) | 0.506 | 0.750 |
| T8SFD | 0.954 (0.615 - 1.480) | 0.834 | 0.929 | L2SFA | 0.767 (0.500 - 1.176) | 0.225 | 0.598 |
| T8SFI | 0.873 (0.533 - 1.431) | 0.591 | 0.761 | L2SFD | 1.271 (0.854 - 1.889) | 0.237 | 0.598 |
| T8SMA | 0.681 (0.445 - 1.042) | 0.076 | 0.499 | L2SFI | 0.801 (0.528 - 1.217) | 0.299 | 0.637 |
| T8SMI | 0.807 (0.527 - 1.238) | 0.326 | 0.637 | L2VFA | 0.928 (0.612 - 1.406) | 0.723 | 0.838 |
| T8IMFD | 0.842 (0.533 - 1.331) | 0.462 | 0.715 | L2VFD | 1.344 (0.878 - 2.056) | 0.174 | 0.598 |
| T9SFA | 0.755 (0.481 - 1.183) | 0.220 | 0.598 | L2VFI | 0.994 (1.506 - 0.656) | 0.977 | 0.995 |
| T9SFD | 1.305 (0.873 - 1.957) | 0.194 | 0.598 | L2SMA | 0.736 (0.494 - 1.098) | 0.133 | 0.541 |
| T9SFI | 0.757 (0.484 - 1.183) | 0.222 | 0.598 | L2SMI | 0.701 (0.459 - 1.071) | 0.100 | 0.499 |
| T9SMA | 0.704 (0.469 - 1.056) | 0.090 | 0.499 | L2IMFD | 1.178 (0.831 - 1.671) | 0.357 | 0.637 |
| **T9SMI** | **0.607 (0.411 - 0.898)** | **0.012*** | 0.253 | L3SFA | 0.686 (0.404 - 1.166) | 0.164 | 0.596 |
| T9IMFD | 0.911 (0.622 - 1.335) | 0.634 | 0.768 | L3SFD | 1.174 (0.782 - 1.763) | 0.439 | 0.715 |
| T10SFA | 0.896 (0.579 - 1.385) | 0.621 | 0.764 | L3SFI | 0.758 (0.451 - 1.271) | 0.293 | 0.637 |
| T10SFD | 1.166 (0.780 - 1.742) | 0.454 | 0.715 | L3VFA | 0.826 (0.550 - 1.240) | 0.356 | 0.637 |
| T10SFI | 0.947 (0.612 - 1.468) | 0.808 | 0.923 | L3VFD | 1.124 (0.746 - 1.694) | 0.577 | 0.761 |
| **T10SMA** | **0.612 (0.406 - 0.923)** | **0.019*** | 0.253 | L3VFI | 0.783 (0.515 - 1.192) | 0.253 | 0.598 |
| **T10SMI** | **0.631 (0.424 - 0.938)** | **0.023*** | 0.260 | L3SMA | 0.704 (0.468 - 1.057) | 0.091 | 0.499 |
| T10IMFA | 0.752 (0.522 - 1.082) | 0.124 | 0.541 | **L3SMI** | **0.639 (0.412 - 0.991)** | **0.045*** | 0.327 |
| T10IMFD | 1.352 (0.938 - 1.950) | 0.106 | 0.499 | L3IMFA | 1.343 (0.907 - 1.989) | 0.141 | 0.541 |
| T10IMFI | 0.736 (0.514 - 1.054) | 0.094 | 0.499 | L3IMFD | 1.176 (0.834 - 1.657) | 0.355 | 0.637 |
| T11SFA | 0.794 (0.509 - 1.241) | 0.311 | 0.637 | L3IMFI | 1.350 (0.904 - 2.015) | 0.142 | 0.541 |
| T11SFD | 1.273 (0.850 - 1.907) | 0.241 | 0.598 | L4SFA | 0.765 (0.497 - 1.175) | 0.220 | 0.598 |
| T11SFI | 0.806 (0.515 - 1.263) | 0.346 | 0.637 | L4SFD | 1.114 (0.764 - 1.624) | 0.576 | 0.761 |
| **T11SMA** | **0.614 (0.411 - 0.917)** | **0.017*** | 0.253 | L4SFI | 0.836 (0.535 - 1.307) | 0.432 | 0.715 |
| **T11SMI** | **0.630 (0.415 - 0.956)** | **0.030*** | 0.267 | L4VFA | 0.921 (0.604 - 1.404) | 0.702 | 0.826 |
| T11IMFD | 1.287 (0.865 - 1.916) | 0.213 | 0.598 | L4VFD | 1.269 (0.843 - 1.911) | 0.254 | 0.598 |
| T12SFA | 0.899 (0.570 - 1.418) | 0.649 | 0.775 | L4VFI | 0.881 (0.585 - 1.326) | 0.543 | 0.759 |
| T12SFD | 1.193 (0.797 - 1.788) | 0.391 | 0.666 | L4IMFD | 1.233 (0.870 - 1.747) | 0.239 | 0.598 |
| T12SFI | 0.812 (0.516 - 1.277) | 0.366 | 0.637 | L5SFA | 0.991 (0.646 - 1.520) | 0.967 | 0.995 |
| **T12SMA** | **0.617 (0.411 - 0.925)** | **0.019*** | 0.253 | L5SFD | 1.244 (0.828 - 1.869) | 0.294 | 0.637 |
| **T12SMI** | **0.650 (0.432 - 0.977)** | **0.038*** | 0.304 | L5SFI | 0.998 (0.652 - 1.527) | 0.992 | 0.995 |
| T12IMFD | 1.105 (0.770 - 1.585) | 0.588 | 0.761 | L5VFA | 0.875 (0.579 - 1.323) | 0.526 | 0.754 |
| L1SFA | 0.890 (0.568 - 1.397) | 0.613 | 0.764 | L5VFD | 1.162 (0.769 - 1.756) | 0.477 | 0.720 |
| L1SFD | 1.042 (0.705 - 1.541) | 0.836 | 0.929 | L5VFI | 1.001 (0.652 - 1.536) | 0.995 | 0.995 |
| L1SFI | 0.885 (0.562 - 1.395) | 0.599 | 0.761 | L5SMA | 0.986 (0.661 - 1.471) | 0.945 | 0.995 |
| L1VFA | 0.961 (0.625 - 1.475) | 0.853 | 0.935 | L5SMI | 1.134 (0.750 - 1.715) | 0.550 | 0.759 |
| L1VFD | 0.819 (0.532 - 1.261) | 0.364 | 0.637 | **L5IMFD** | **1.515 (1.051 - 2.185)** | **0.026*** | 0.260 |
| L1VFI | 0.993 (0.654 - 1.508) | 0.975 | 0.995 | SFV | 0.869 (0.561 - 1.346) | 0.528 | 0.754 |
| **L1SMA** | **0.505 (0.338 - 0.755)** | **<0.001**** | 0.072 | VFV | 1.028 (0.677 - 1.563) | 0.898 | 0.971 |
| **L1SMI** | **0.578 (0.384 - 0.868)** | **0.008*** | 0.253 | SMV | 1.164 (0.775 - 1.748) | 0.465 | 0.715 |

Note. Each body composition parameter was analyzed separately in an individual multivariable Cox regression model. HR was adjusted with age, BMI Grade, Stage and Her2 (Low group as reference).

## Table S8. Multivariable cox analyses of body composition in male surgery cohorts (adjusted for CEA, CA199, Grade, Stage, and Her2)

| **Parameters** | **HR (95%CI)** | ***P* value** | **ad *P* value** | **Parameters** | **HR (95%CI)** | ***P* value** | **ad *P* value** |
| --- | --- | --- | --- | --- | --- | --- | --- |
| T8SFA | 0.740 (0.477 - 1.149) | 0.180 | 0.351 | L1IMFD | 1.081 (0.748 - 1.563) | 0.680 | 0.702 |
| T8SFD | 1.137 (0.734 - 1.761) | 0.564 | 0.645 | L2SFA | 0.786 (0.537 - 1.151) | 0.215 | 0.366 |
| T8SFI | 0.782 (0.493 - 1.242) | 0.299 | 0.449 | L2SFD | 1.385 (0.948 - 2.023) | 0.092 | 0.283 |
| **T8SMA** | **0.551 (0.354 - 0.859)** | **0.008*** | 0.091 | L2SFI | 0.831 (0.565 - 1.220) | 0.344 | 0.459 |
| T8SMI | 0.665 (0.417 - 1.055) | 0.083 | 0.277 | L2VFA | 0.825 (0.564 - 1.209) | 0.325 | 0.459 |
| T8IMFD | 0.913 (0.582 - 1.433) | 0.693 | 0.702 | **L2VFD** | **1.581 (1.042 - 2.397)** | **0.031*** | 0.191 |
| T9SFA | 0.746 (0.496 - 1.122) | 0.160 | 0.328 | L2VFI | 0.864 (0.590 - 1.266) | 0.454 | 0.556 |
| **T9SFD** | **1.529 (1.015 - 2.304)** | **0.042*** | 0.207 | L2SMA | 0.750 (0.514 - 1.094) | 0.135 | 0.288 |
| T9SFI | 0.724 (0.481 - 1.089) | 0.121 | 0.288 | L2SMI | 0.675 (0.453 - 1.006) | 0.053 | 0.223 |
| **T9SMA** | **0.644 (0.423 - 0.978)** | **0.039*** | 0.207 | L2IMFD | 1.363 (0.928 - 2.002) | 0.115 | 0.288 |
| **T9SMI** | **0.552 (0.367 - 0.830)** | **0.004*** | 0.091 | L3SFA | 0.673 (0.419 - 1.083) | 0.103 | 0.287 |
| T9IMFD | 1.080 (0.738 - 1.581) | 0.693 | 0.702 | L3SFD | 1.407 (0.950 - 2.084) | 0.089 | 0.283 |
| T10SFA | 0.863 (0.577 - 1.290) | 0.473 | 0.556 | L3SFI | 0.668 (0.411 - 1.087) | 0.104 | 0.287 |
| T10SFD | 1.344 (0.910 - 1.988) | 0.137 | 0.288 | L3VFA | 0.816 (0.564 - 1.182) | 0.284 | 0.437 |
| T10SFI | 0.907 (0.602 - 1.366) | 0.641 | 0.693 | L3VFD | 1.215 (0.824 - 1.791) | 0.325 | 0.459 |
| **T10SMA** | **0.583 (0.394 - 0.864)** | **0.007*** | 0.091 | L3VFI | 0.760 (0.514 - 1.122) | 0.167 | 0.334 |
| **T10SMI** | **0.588 (0.397 - 0.871)** | **0.008*** | 0.091 | **L3SMA** | **0.666 (0.448 - 0.989)** | **0.044*** | 0.207 |
| T10IMFA | 0.751 (0.518 - 1.088) | 0.130 | 0.288 | **L3SMI** | **0.631 (0.416 - 0.957)** | **0.030*** | 0.191 |
| T10IMFD | 1.420 (0.977 - 2.066) | 0.066 | 0.240 | **L3IMFA** | **1.580 (1.073 - 2.327)** | **0.020*** | 0.145 |
| T10IMFI | 0.833 (0.566 - 1.225) | 0.352 | 0.462 | L3IMFD | 1.179 (0.815 - 1.705) | 0.381 | 0.476 |
| T11SFA | 0.775 (0.518 - 1.159) | 0.214 | 0.366 | **L3IMFI** | **1.638 (1.107 - 2.424)** | **0.014*** | 0.140 |
| T11SFD | 1.379 (0.940 - 2.023) | 0.101 | 0.287 | L4SFA | 0.774 (0.523 - 1.148) | 0.204 | 0.366 |
| T11SFI | 0.815 (0.539 - 1.233) | 0.333 | 0.459 | L4SFD | 1.248 (0.855 - 1.821) | 0.251 | 0.410 |
| **T11SMA** | **0.596 (0.409 - 0.867)** | **0.007*** | 0.091 | L4SFI | 0.766 (0.514 - 1.142) | 0.191 | 0.364 |
| **T11SMI** | **0.620 (0.418 - 0.921)** | **0.018*** | 0.145 | L4VFA | 0.901 (0.609 - 1.332) | 0.599 | 0.675 |
| **T11IMFD** | **1.610 (1.007 - 2.571)** | **0.047*** | 0.209 | L4VFD | 1.353 (0.917 - 1.996) | 0.128 | 0.288 |
| T12SFA | 0.858 (0.570 - 1.292) | 0.463 | 0.556 | L4VFI | 0.820 (0.563 - 1.196) | 0.303 | 0.449 |
| T12SFD | 1.426 (0.957 - 2.123) | 0.081 | 0.277 | L4IMFD | 1.328 (0.917 - 1.924) | 0.133 | 0.288 |
| T12SFI | 0.866 (0.573 - 1.307) | 0.493 | 0.572 | L5SFA | 0.834 (0.600 - 1.161) | 0.283 | 0.437 |
| **T12SMA** | **0.634 (0.433 - 0.927)** | **0.019*** | 0.145 | L5SFD | 1.297 (0.926 - 1.818) | 0.130 | 0.288 |
| T12SMI | 0.693 (0.472 - 1.018) | 0.062 | 0.236 | L5SFI | 0.807 (0.568 - 1.145) | 0.230 | 0.383 |
| T12IMFD | 1.292 (0.872 - 1.916) | 0.202 | 0.366 | L5VFA | 0.760 (0.539 - 1.071) | 0.117 | 0.288 |
| L1SFA | 0.860 (0.574 - 1.289) | 0.466 | 0.556 | L5VFD | 1.240 (0.888 - 1.729) | 0.206 | 0.366 |
| L1SFD | 1.198 (0.827 - 1.735) | 0.340 | 0.459 | L5VFI | 0.848 (0.592 - 1.215) | 0.370 | 0.470 |
| L1SFI | 0.827 (0.552 - 1.239) | 0.358 | 0.462 | L5SMA | 0.909 (0.612 - 1.348) | 0.636 | 0.693 |
| L1VFA | 0.918 (0.619 - 1.362) | 0.674 | 0.702 | L5SMI | 1.017 (0.658 - 1.572) | 0.937 | 0.937 |
| L1VFD | 1.512 (0.979 - 2.336) | 0.062 | 0.236 | **L5IMFD** | **1.489 (1.024 - 2.167)** | **0.037*** | 0.207 |
| L1VFI | 0.810 (0.552 - 1.188) | 0.280 | 0.437 | SFV | 0.826 (0.558 - 1.224) | 0.342 | 0.459 |
| **L1SMA** | **0.526 (0.359 - 0.771)** | **< 0.001**** | 0.080 | VFV | 0.918 (0.632 - 1.335) | 0.655 | 0.699 |
| **L1SMI** | **0.584 (0.397 - 0.859)** | **0.006*** | 0.091 | SMV | 0.915 (0.632 - 1.325) | 0.638 | 0.693 |

Note. Each body composition parameter was analyzed separately in an individual multivariable Cox regression model. HR was adjusted with CEA, CA199, Grade, Stage and Her2 (Low group as reference).

## Table S9. Multivariable cox analyses of body composition in male surgery cohorts (adjusted for age, BMI, CEA, CA199, Grade, Stage, and Her2)

| **Parameters** | **HR (95%CI)** | ***P* value** | **ad *P* value** | **Parameters** | **HR (95%CI)** | ***P* value** | **ad *P* value** |
| --- | --- | --- | --- | --- | --- | --- | --- |
| T8SFA | 0.718 (0.411 - 1.256) | 0.246 | 0.621 | L1IMFD | 0.994 (0.682 - 1.449) | 0.974 | 0.983 |
| T8SFD | 1.031 (0.627 - 1.696) | 0.904 | 0.939 | L2SFA | 0.825 (0.511 - 1.333) | 0.432 | 0.679 |
| T8SFI | 0.785 (0.444 - 1.389) | 0.406 | 0.679 | L2SFD | 1.277 (0.824 - 1.978) | 0.274 | 0.621 |
| T8SMA | 0.659 (0.406 - 1.070) | 0.092 | 0.549 | L2SFI | 0.905 (0.568 - 1.443) | 0.676 | 0.829 |
| T8SMI | 0.785 (0.478 - 1.287) | 0.337 | 0.657 | L2VFA | 0.909 (0.575 - 1.439) | 0.684 | 0.829 |
| T8IMFD | 0.946 (0.570 - 1.572) | 0.831 | 0.912 | L2VFD | 1.523 (0.942 - 2.464) | 0.086 | 0.549 |
| T9SFA | 0.728 (0.436 - 1.218) | 0.227 | 0.621 | L2VFI | 0.956 (0.602 - 1.517) | 0.847 | 0.912 |
| T9SFD | 1.471 (0.933 - 2.315) | 0.097 | 0.549 | L2SMA | 0.911 (0.589 - 1.412) | 0.679 | 0.829 |
| T9SFI | 0.729 (0.438 - 1.215) | 0.219 | 0.621 | L2SMI | 0.803 (0.504 - 1.277) | 0.353 | 0.657 |
| T9SMA | 0.776 (0.492 - 1.224) | 0.275 | 0.621 | L2IMFD | 1.249 (0.846 - 1.844) | 0.264 | 0.621 |
| **T9SMI** | **0.598 (0.388 - 0.920)** | **0.019*** | 0.549 | L3SFA | 0.641 (0.367 - 1.122) | 0.120 | 0.600 |
| T9IMFD | 0.995 (0.651 - 1.522) | 0.983 | 0.983 | L3SFD | 1.357 (0.853 - 2.158) | 0.197 | 0.621 |
| T10SFA | 0.923 (0.561 - 1.520) | 0.751 | 0.885 | L3SFI | 0.620 (0.350 - 1.096) | 0.100 | 0.549 |
| T10SFD | 1.259 (0.803 - 1.980) | 0.315 | 0.630 | L3VFA | 0.831 (0.529 - 1.305) | 0.420 | 0.679 |
| T10SFI | 0.938 (0.569 - 1.546) | 0.802 | 0.912 | L3VFD | 1.166 (0.736 - 1.848) | 0.512 | 0.759 |
| T10SMA | 0.684 (0.436 - 1.073) | 0.098 | 0.549 | L3VFI | 0.807 (0.509 - 1.282) | 0.365 | 0.658 |
| **T10SMI** | **0.645 (0.419 - 0.991)** | **0.045*** | 0.549 | L3SMA | 0.814 (0.518 - 1.277) | 0.370 | 0.658 |
| T10IMFA | 0.713 (0.476 - 1.071) | 0.103 | 0.549 | L3SMI | 0.718 (0.440 - 1.174) | 0.187 | 0.621 |
| T10IMFD | 1.422 (0.945 - 2.141) | 0.091 | 0.549 | L3IMFA | 1.381 (0.900 - 2.119) | 0.140 | 0.621 |
| T10IMFI | 0.804 (0.541 - 1.196) | 0.283 | 0.621 | L3IMFD | 1.100 (0.756 - 1.599) | 0.619 | 0.829 |
| T11SFA | 0.784 (0.472 - 1.304) | 0.349 | 0.657 | L3IMFI | 0.684 (0.442 - 1.060) | 0.090 | 0.549 |
| T11SFD | 1.274 (0.816 - 1.989) | 0.287 | 0.621 | L4SFA | 0.778 (0.478 - 1.263) | 0.308 | 0.630 |
| T11SFI | 0.799 (0.480 - 1.330) | 0.387 | 0.673 | L4SFD | 1.121 (0.735 - 1.709) | 0.597 | 0.823 |
| T11SMA | 0.671 (0.437 - 1.028) | 0.067 | 0.549 | L4SFI | 0.761 (0.463 - 1.250) | 0.281 | 0.621 |
| T11SMI | 0.658 (0.428 - 1.011) | 0.056 | 0.549 | L4VFA | 0.956 (0.592 - 1.546) | 0.855 | 0.912 |
| T11IMFD | 1.359 (0.863 - 2.141) | 0.185 | 0.621 | L4VFD | 1.303 (0.821 - 2.070) | 0.262 | 0.621 |
| T12SFA | 0.856 (0.509 - 1.439) | 0.558 | 0.797 | L4VFI | 0.831 (0.522 - 1.321) | 0.433 | 0.679 |
| T12SFD | 1.310 (0.836 - 2.053) | 0.238 | 0.621 | L4IMFD | 1.172 (0.800 - 1.717) | 0.416 | 0.679 |
| T12SFI | 0.838 (0.500 - 1.406) | 0.505 | 0.759 | L5SFA | 0.952 (0.585 - 1.550) | 0.844 | 0.912 |
| T12SMA | 0.736 (0.474 - 1.143) | 0.172 | 0.621 | L5SFD | 1.299 (0.818 - 2.063) | 0.268 | 0.621 |
| T12SMI | 0.762 (0.489 - 1.188) | 0.230 | 0.621 | L5SFI | 0.946 (0.585 - 1.531) | 0.822 | 0.912 |
| T12IMFD | 1.103 (0.736 - 1.653) | 0.634 | 0.829 | L5VFA | 0.836 (0.527 - 1.327) | 0.447 | 0.688 |
| L1SFA | 0.898 (0.537 - 1.502) | 0.683 | 0.829 | L5VFD | 1.125 (0.705 - 1.795) | 0.622 | 0.829 |
| L1SFD | 1.073 (0.694 - 1.658) | 0.752 | 0.885 | L5VFI | 0.992 (0.613 - 1.605) | 0.974 | 0.983 |
| L1SFI | 0.861 (0.512 - 1.447) | 0.571 | 0.801 | L5SMA | 1.261 (0.806 - 1.972) | 0.309 | 0.630 |
| L1VFA | 0.942 (0.579 - 1.531) | 0.809 | 0.912 | L5SMI | 1.325 (0.825 - 2.128) | 0.245 | 0.621 |
| L1VFD | 1.407 (0.848 - 2.333) | 0.186 | 0.621 | **L5IMFD** | **1.493 (1.006 - 2.214)** | **0.046*** | 0.549 |
| L1VFI | 0.903 (0.566 - 1.441) | 0.670 | 0.829 | SFV | 0.859 (0.531 - 1.393) | 0.538 | 0.783 |
| **L1SMA** | **0.588 (0.382 - 0.907)** | **0.016*** | 0.549 | VFV | 1.038 (0.664 - 1.623) | 0.870 | 0.916 |
| L1SMI | 0.651 (0.416 - 1.017) | 0.059 | 0.549 | SMV | 1.280 (0.829 - 1.980) | 0.265 | 0.621 |

Note. Each body composition parameter was analyzed separately in an individual multivariable Cox regression model. HR was adjusted with age, BMI, CEA, CA199, Grade, Stage and Her2 (Low group as reference).

## Table S10. Multivariable cox analyses of body composition in female surgery cohorts (adjusted for age, BMI, Grade, Stage, Her2)

| **Parameters** | **HR (95%CI)** | ***P* value** | **ad *P* value** | **HR# (95%CI)** | ***P#* value** | **ad *P#* value** |
| --- | --- | --- | --- | --- | --- | --- |
| T10IMFD | 1.184 (0.764 - 1.834) | 0.450 | 0.450 | 1.240 (0.767 - 2.005) | 0.380 | 0.489 |
| T11IMFD | 1.472 (0.937 - 2.313) | 0.093 | 0.152 | 1.311 (0.824 - 2.085) | 0.254 | 0.381 |
| **L1VFD** | **1.748 (1.122 - 2.717)** | **0.014*** | 0.063 | **2.132 (1.290 - 3.521)** | **0.003*** | **0.027*** |
| L2SFD | 1.294 (0.773 - 2.165) | 0.327 | 0.368 | 1.190 (0.706 - 2.008) | 0.513 | 0.577 |
| L2VFD | 1.655 (0.999 - 2.451) | 0.051 | 0.152 | **1.919 (1.130 - 3.268)** | **0.016*** | 0.072 |
| L3SFD | 1.300 (0.840 - 2.012) | 0.239 | 0.307 | 1.148 (0.706 - 1.866) | 0.577 | 0.577 |
| L3VFD | 1.441 (1.441 - 2.227) | 0.101 | 0.152 | 1.647 (0.994 - 2.732) | 0.053 | 0.103 |
| **L4SMA** | **0.514 (0.331 - 0.798)** | **0.003*** | **0.027*** | 0.617 (0.375 - 1.015) | 0.057 | 0.103 |
| L4VFD | 1.493 (0.927 - 2.410) | 0.100 | 0.152 | 1.664 (0.995 - 2.786) | 0.052 | 0.103 |

Note. Each body composition parameter was analyzed separately in an individual multivariable Cox regression model. HR was adjusted with Grade, Stage and Her2; HR# was adjusted with age, BMI, Grade, Stage and Her2.

## Table S11. Multivariable cox analyses of body composition in female surgery cohorts (adjusted for age, BMI, CEA, CA199, Grade, Stage, Her2)

| **Parameters** | **HR (95%CI)** | ***P* value** | **ad *P* value** | **HR# (95%CI)** | ***P#* value** | **ad *P#* value** |
| --- | --- | --- | --- | --- | --- | --- |
| T10IMFD | 1.277 (0.778 - 2.096) | 0.333 | 0.333 | 1.301 (0.758 - 2.233) | 0.339 | 0.381 |
| T11IMFD | 1.551 (0.928 - 2.592) | 0.094 | 0.206 | 1.375 (0.809 - 2.336) | 0.240 | 0.360 |
| L1VFD | 1.57 (0.936 - 2.639) | 0.087 | 0.206 | 1.883 (1.020 - 3.472) | **0.043*** | 0.194 |
| L2SFD | 1.46 (0.805 - 2.667) | 0.211 | 0.271 | 1.379 (0.754 - 2.525) | 0.297 | 0.381 |
| L2VFD | 1.631 (0.952- 2.801) | 0.075 | 0.206 | 2.105 (1.094 - 4.049) | **0.026*** | 0.194 |
| L3SFD | 1.285 (0.772 - 2.141) | 0.333 | 0.333 | 1.048 (0.592 - 1.859) | 0.871 | 0.871 |
| L3VFD | 1.481 (0.883 - 2.488) | 0.137 | 0.206 | 1.783 (0.957 - 3.322) | 0.069 | 0.198 |
| **L4SMA** | **0.497 (0.295 - 0.836)** | **0.008*** | 0.072 | 0.627 (0.351 - 1.119) | 0.114 | 0.205 |
| L4VFD | 1.569 (0.887 - 2.778) | 0.121 | 0.206 | 1.712 (0.924 - 3.175) | 0.088 | 0.198 |

Note. Each body composition parameter was analyzed separately in an individual multivariable Cox regression model. HR was adjusted with CEA, CA199, Grade, Stage and Her2; HR# was adjusted with age, BMI, CEA, CA199, Grade, Stage and Her2.

## Table S12. Multivariable cox analyses of body composition in female ICI cohorts (adjusted for age, BMI, Grade, Stage, Her2, PDL1 CPS)

| **Parameters** | **HR (95%CI)** | **P value** | **ad *P* value** | **HR# (95%CI)** | **P# value** | **ad *P#* value** |
| --- | --- | --- | --- | --- | --- | --- |
| **T8SFI** | **0.925 (0.871 - 0.983)** | **0.012*** | **0.045*** | **0.910 (0.845 - 0.981)** | **0.013*** | **0.042*** |
| T9SFD | 1.037 (0.995 - 1.081) | 0.084 | 0.107 | 1.030 (0.979 - 1.085) | 0.252 | 0.282 |
| T12SFA | 0.974 (0.945 - 1.003) | 0.081 | 0.107 | 0.971 (0.933 - 1.012) | 0.163 | 0.237 |
| T12SFI | 0.929 (0.858 - 1.005) | 0.068 | 0.107 | 0.916 (0.820 - 1.024) | 0.123 | 0.197 |
| **L1SFA** | **0.972 (0.947 - 0.998)** | **0.036*** | 0.068 | **0.961 (0.925 - 0.999)** | **0.045*** | 0.080 |
| L1SFD | 1.043 (0.993 - 1.095) | 0.093 | 0.107 | 1.036 (0.974 - 1.102) | 0.264 | 0.282 |
| **L1SFI** | **0.925 (0.862 - 0.992)** | **0.029*** | 0.064 | **0.892 (0.806 - 0.987)** | **0.028*** | 0.073 |
| **L2SFA** | **0.972 (0.951 - 0.995)** | **0.015*** | **0.045*** | **0.964 (0.936 - 0.992)** | **0.013*** | **0.042*** |
| L2SFD | 1.048 (0.992 - 1.106) | 0.092 | 0.107 | 1.040 (0.976 - 1.108) | 0.230 | 0.282 |
| **L2SFI** | **0.926 (0.872 - 0.983)** | **0.012*** | **0.045*** | **0.901 (0.834 - 0.974)** | **0.008*** | **0.042*** |
| **L3SFA** | **0.957 (0.929 - 0.986)** | **0.003*** | **0.023*** | **0.942 (0.902 - 0.983)** | **0.006*** | **0.042*** |
| **L3SFI** | **0.889 (0.823 - 0.961)** | **0.003*** | **0.023*** | **0.851 (0.761 - 0.951)** | **0.005*** | **0.042*** |
| L3IMFD | 1.043 (0.966 - 1.127) | 0.283 | 0.283 | 1.049 (0.965 - 1.140) | 0.261 | 0.282 |
| **L4SFA** | **0.976 (0.954 - 0.998)** | **0.030*** | 0.064 | **0.970 (0.944 - 0.997)** | **0.032*** | 0.073 |
| L4SFD | 1.041 (0.973 - 1.115) | 0.241 | 0.258 | 1.033 (0.954 - 1.118) | 0.429 | 0.429 |
| **L4SFI** | **0.940 (0.889 - 0.995)** | **0..033*** | 0.064 | **0.928 (0.865 - 0.996)** | **0.038*** | 0.076 |

Note. Each body composition parameter was analyzed separately in an individual multivariable Cox regression model. HR was adjusted with Grade, Stage, Her2 and PDL1 CPS; HR# was adjusted with age, BMI, Grade, Stage, Her2 and PDL1 CPS.

## Table S13. Multivariable cox analyses of body composition in female ICI cohorts (adjusted for age, BMI, CEA, CA199, Grade, Stage, Her2, PDL1 CPS)

| **Parameters** | **HR (95%CI)** | **P value** | **ad *P* value** | **HR# (95%CI)** | **P# value** | **ad *P#* value** |
| --- | --- | --- | --- | --- | --- | --- |
| **T8SFI** | **0.911 (0.851 - 0.976)** | **0.008*** | **0.022*** | **0.860 (0.757 - 0.976)** | **0.020*** | 0.066 |
| **T9SFD** | **1.065 (1.015 - 1.118)** | **0.011*** | **0.022*** | 1.056 (0.991 - 1.125) | 0.094 | 0.117 |
| T12SFA | 0.971 (0.941 - 1.002) | 0.067 | 0.077 | 0.984 (0.935 - 1.036) | 0.548 | 0.548 |
| T12SFI | 0.917 (0.841 - 1.000) | 0.050 | 0.062 | 0.938 (0.814 - 1.080) | 0.373 | 0.398 |
| **L1SFA** | **0.969 (0.943 - 0.997)** | **0.030*** | **0.044*** | 0.956 (0.899 - 1.016) | 0.148 | 0.169 |
| **L1SFD** | **1.071 (1.012 - 1.133)** | **0.017*** | **0.030*** | 1.070 (0.989 - 1.157) | 0.093 | 0.117 |
| **L1SFI** | **0.913 (0.845 - 0.987)** | **0.022*** | **0.035*** | 0.846 (0.705 - 1.016) | 0.074 | 0.117 |
| **L2SFA** | **0.966 (0.941 - 0.992)** | **0.010*** | **0.022*** | **0.947 (0.902 - 0.996)** | **0.033*** | 0.066 |
| **L2SFD** | **1.063 (1.002 - 1.128)** | **0.042*** | 0.056 | 1.070 (0.988 - 1.159) | 0.095 | 0.117 |
| **L2SFI** | **0.903 (0.839 - 0.972)** | **0.007*** | **0.022*** | **0.835 (0.719 - 0.968)** | **0.017*** | 0.066 |
| **L3SFA** | **0.943 (0.909 - 0.978)** | **0.003*** | **0.022*** | **0.897 (0.827 - 0.973)** | **0.009*** | 0.066 |
| **L3SFI** | **0.846 (0.767 - 0.934)** | **< 0.001*** | **0.016*** | **0.714 (0.556 - 0.916)** | **0.008*** | 0.066 |
| L3IMFD | 1.069 (0.992 - 1.151) | 0.079 | 0.084 | **1.155 (1.014 - 1.316)** | **0.030*** | 0.066 |
| **L4SFA** | **0.960 (0.931 - 0.991)** | **0.011*** | **0.022*** | **0.948 (0.905 - 0.993)** | **0.025*** | 0.066 |
| L4SFD | 1.057 (0.987 - 1.132) | 0.113 | 0.113 | 1.104 (0.990 - 1.232) | 0.075 | 0.117 |
| **L4SFI** | **0.899 (0.832 - 0.972)** | **0.007*** | **0.022*** | **0.887 (0.795 - 0.988)** | **0.030*** | 0.066 |

Note. Each body composition parameter was analyzed separately in an individual multivariable Cox regression model. HR was adjusted with CEA, CA199, Grade, Stage, Her2 and PDL1 CPS; HR# was adjusted with age, BMI, CEA, CA199, Grade, Stage, Her2 and PDL1 CPS.
